# Recent Progress on Low-Temperature Selective Catalytic Reduction of NO_x_ with Ammonia

**DOI:** 10.3390/molecules29184506

**Published:** 2024-09-23

**Authors:** Eun Duck Park

**Affiliations:** 1 Department of Energy Systems Research, Ajou University, Suwon 16499, Republic of Korea; edpark@ajou.ac.kr; Tel.: +82-31-219-2384; 2 Department of Chemical Engineering, Ajou University, Suwon 16499, Republic of Korea

**Keywords:** low-temperature selective catalytic reduction of NO with NH_3_ (NH_3_-SCR), catalyst, NO_x_ reduction, SO_2_/H_2_O tolerance, transition metal-based catalysts

## Abstract

Selective catalytic reduction of nitrogen oxides (NO_x_) with ammonia (NH_3_-SCR) has been implemented in response to the regulation of NO_x_ emissions from stationary and mobile sources above 300 °C. However, the development of NH_3_-SCR catalysts active at low temperatures below 200 °C is still needed to improve the energy efficiency and to cope with various fuels. In this review article, recent reports on low-temperature NH_3_-SCR catalysts are systematically summarized. The redox property as well as the surface acidity are two main factors that affect the catalytic activity. The strong redox property is beneficial for the low-temperature NH_3_-SCR activity but is responsible for N_2_O formation. The multiple electron transfer system is more plausible for controlling redox properties. H_2_O and SO_x_, which are often found with NO_x_ in flue gas, have a detrimental effect on NH_3_-SCR activity, especially at low temperatures. The competitive adsorption of H_2_O can be minimized by enhancing the hydrophobic property of the catalyst. Various strategies to improve the resistance to SO_x_ poisoning are also discussed.

## 1. Introduction

Anthropogenic pollutant emissions increased with human activity until a few decades ago, posing a threat to human well-being. However, over the past few decades, regulations on the emission of these pollutants and the development of technologies to control emissions have had some success in preventing significant pollution. Sulfur oxides (SO_x_), nitrogen oxides (NO_x_ and N_2_O), CO, and volatile organic compounds (VOCs) are representative gaseous pollutants that are now strictly regulated [1]. In addition, greenhouse gases, including CO_2_ and methane, are currently or will be regulated in the near future, depending on the country.

NO_x_, such as nitrogen monoxide (NO) and nitrogen dioxide (NO_2_), is formed from a variety of stationary and mobile sources. Fossil fuel-based power plants and internal combustion engine-based transportation vehicles are prime examples of stationary and mobile sources of NO_x_ emissions, respectively. In any case, thermal NO_x_, formed at high temperatures when nitrogen oxidizes with oxygen in the air, is the primary pathway for NO_x_ emissions. As conventional power plants and internal combustion engine-based vehicles are replaced with renewable energy and electric vehicles, respectively, to cope with the CO_2_ emissions problem, the NO_x_ emissions are expected to continue to decline. Meanwhile, with ammonia gaining much attention as a non-carbon fuel, fuel NO_x_, which is formed through the partial oxidation of ammonia, can be another pathway for NO_x_ emissions in the stationary sources [2,3,4].

The selective catalytic reduction of NO_x_ with ammonia (NH_3_-SCR) is the most widely adopted method for controlling NO_x_ emissions from stationary sources among the various methods developed to date [5,6]. The following reactions occur during NH_3_-SCR in the absence of oxygen.

6NO + 4NH_3_ → 5N_2_ + 6H_2_O (1)

6NO_2_ + 8NH_3_ → 7N_2_ + 12H_2_O (2)

NO + NO_2_ + 2NH_3_ → 2N_2_ + 3H_2_O (3)

In practice, because most flue gases contain varying concentrations of oxygen, the following reactions take place.

4NO + 4NH_3_ + O_2_ → 4N_2_ + 6H_2_O (4)

2NO + 4NH_3_ + 2O_2_ →3N_2_ + 6H_2_O (5)

2NO_2_ + 4NH_3_ + O_2_ → 3N_2_ + 6H_2_O (6)

In addition, ammonia oxidations, shown below, can occur in the presence of oxygen, which is not favorable for NH_3_-SCR.

4NH_3_ + 3O_2_ → 2N_2_ + 6H_2_O (7)

4NH_3_ + 5O_2_ → 4NO + 6H_2_O (8)

4NH_3_ + 7O_2_ → 4NO_2_ + 6H_2_O (9)

2NH_3_ + 2O_2_ → N_2_O + 3H_2_O (10)

N_2_O can be additionally formed via the following reaction in the presence of the catalyst.

4NO + 4NH_3_ + 3O_2_ → 4N_2_O + 6H_2_O (11)

Metal (W and/or Mo) oxides-promoted V_2_O_5_/TiO_2_ is a representative commercial catalyst for NH_3_-SCR, achieving high NO_x_ conversion over a wide operating temperature range of 300–400 °C [7,8,9]. Because flue gas composition varies depending on the fuel (e.g., coal, biomass, organic wastes, oil, natural gas, etc.), the NH_3_-SCR unit is placed in different locations in the flue gas treatment process depending on the process characteristics [10]. Generally, since the flue gas contains fly ash, impurities, and SO_2_, the dust removal device (e.g., electrostatic precipitator) and the flue gas desulfurization device can be installed to remove the fly ash and SO_2_ in the flue gas, respectively [11]. After these units, additional preheaters are required to raise the flue gas temperature to the proper operating temperature of vanadia-based commercial catalysts because the flue gas temperature is reduced below 200 °C. Therefore, low-temperature NH_3_-SCR catalysts need to be developed to eliminate the increased operating cost and an extra capital cost due to these additional preheating units. 

To date, a variety of NH_3_-SCR catalysts [12,13,14,15,16,17,18,19,20,21,22,23], including Cu-based [24], Ce-based [25,26,27,28], Mn-based [29,30,31], Ba-based [32], and carbon materials-supported catalysts [33,34], have been studied that are active below 200 °C. Compared with NH_3_-SCR catalysts operating at medium and high temperatures, low-temperature NH_3_-SCR catalysts are susceptible to poisoning by water vapor and SO_2_ in flue gas [19,21]. In particular, SO_2_ in flue gas can be catalytically oxidized to form SO_3_, which reacts with ammonia and then converts to ammonium salts (NH_4_HSO_4_ (ABS) and (NH_4_)_2_SO_4_ (AS)), which can block the active sites of NH_3_-SCR [11,35]. Furthermore, additional side reactions forming N_2_O have been observed over these catalysts. Low-temperature NH_3_-SCR catalysts capable of solving the above problems have not yet been reported. This review article summarizes recent progress on low-temperature NH_3_-SCR catalysis and briefly discusses research directions to address the current obstacles.

## 2. Low-Temperature NH_3_-SCR Catalysts

**Figure 1:**
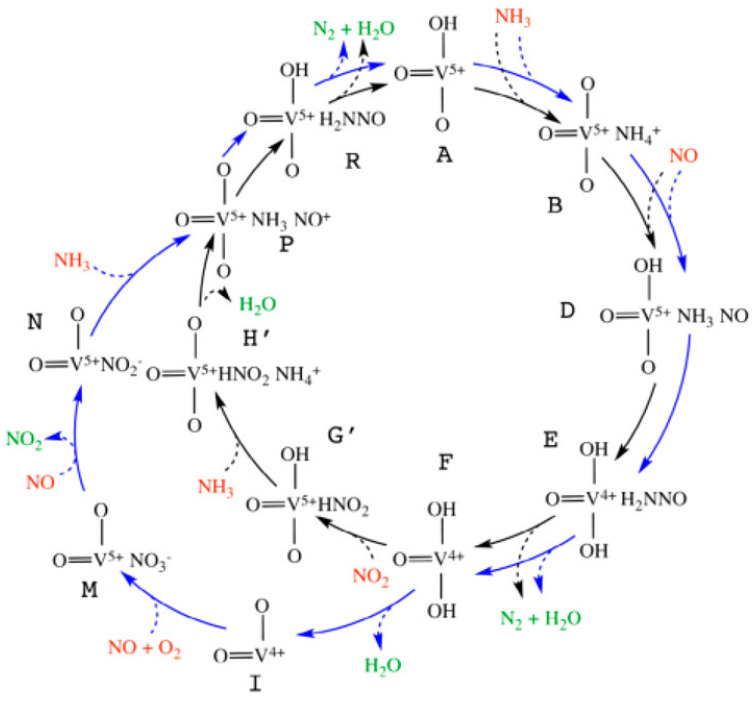
Proposed reaction mechanism over the VO_x_/TiO_2_(001) model. The black circle represents the ‘Fast NH_3_-SCR’ reaction, and the blue circle represents the NO-activation cycle; the ‘Standard NH_3_-SCR’ reaction is the sum of the black and blue cycles. This schematic diagram is reprinted with permission from ref [36]. Copyright 2017 Elsevier.

Vanadia-based catalysts active at moderate temperatures can provide meaningful information about low-temperature NH_3_-SCR catalysis. Arnarson et al. [36] proposed a reaction mechanism of ‘Standard NH_3_-SCR’ combining with ‘Fast NH_3_-SCR’ on a VO_x_/TiO_2_(001) catalyst model (Figure 1). The two cycles shared the same reduction part (A → B → D → E → F → P → R → A in Figure 1) but used NO + O_2_ (F → I → M → N → P in Figure 1) and NO_2_ (F → G′ → H′ → P in Figure 1) for the re-oxidation process, respectively. They noted that the rate of formation and desorption of H_2_O is a decisive factor in the ‘Standard NH_3_-SCR’ reaction at low temperatures and that the reaction of NO_2_ with the reduction sites is responsible for accelerating the ‘Fast NH_3_-SCR’ reaction at low temperatures [36].

The reaction mechanisms [36,37,38] over V_2_O_5_/TiO_2_ catalyst reveal that NH_3_-SCR activity depends on a number of factors, including the redox property of vanadium species between V^+5^ and V^+4^, Brønsted and Lewis acid sites on the catalyst surface, dispersion of vanadium species, acidic and basic properties of the support, contribution of a support to the redox property of vanadium species, the surface hydrophobicity of the support, the hydrothermal stability of the support, and adsorption property of SO_x_. Therefore, these factors should also be considered when designing low-temperature NH_3_-SCR catalysts. First of all, the redox properties of the active metal oxides are crucial for this reaction, so Mn, Fe, Co, Cu, and Ce can be selected as promising candidates for the active metal because they have a variety of metal oxides with different oxidation states and are interconvertible under reaction conditions. However, cobalt oxide can be excluded as a promising candidate for this reaction because of its very high activity towards complete oxidation [39]. Therefore, Cu-, Fe-, Mn-, and Ce-based low-temperature NH_3_-SCR catalysts are covered in the following sections.

### 2.1. Cu-Based Catalysts

Cu-containing metal oxides and Cu-based small-pore zeolites have been reported to be active for low- and medium-temperature NH_3_-SCR. Various factors, including support, Cu precursor, promoter, crystal structure, preparation method, and interface engineering between Cu and support, have been considered [24]. Research on Cu-based catalysts has been motivated by their high NH_3_-SCR activity, especially at low temperatures, but their low SO_2_ tolerance is recognized as a significant barrier to field application. Moreover, due to the low hydrothermal stability of Cu-containing metal oxides, recent research has focused on Cu-based small pore zeolites, as shown in Table 1 [40,41,42,43,44,45,46,47,48,49,50,51,52,53,54,55,56,57,58,59,60,61,62,63,64,65,66,67,68,69,70,71,72,73,74,75,76]. Among them, low-temperature NH_3_-SCR activity was reported over Cu-zeolites such as Cu-LTA [41], Cu-ZSM-5 [42,43,44], Cu-SSZ-13 [47], Cu-SSZ-16 [49], Cu-SSZ-52 [51], Cu-SAPO-34 [52], Cu-UZM-35 [56], and Cu-ZJM-7 [57], metal-promoted Cu-zeolites such as Ce-Cu-SAPO-18 [59], Fe/Cu-SSZ-13 [62], CuY-SAPO-34 [63], CuNd/SAPO-34, Cu-Ce-La-SSZ-13 [65], and Cu-Ce-USY [68], and other Cu-based oxides such as Cu/ZrO_2_ [70] and CuAl layered double oxide (LDO) supported on carbon nanotubes (CNTs) [72].

Only a few reports can be found on Cu-containing metal oxides in the last few years. Chen et al. [69] examined the crystal-plane effects of CuO over CuO/TiO_2_ catalysts on NH_3_-SCR and reported that the proportion of Cu^+^ and surface-adsorbed oxygen (O_α_) in CuO(111)/TiO_2_ catalyst was higher than that of CuO(001)/TiO_2_ catalyst, which could facilitate the NH_3_-SCR reactions. Liu et al. [71] reported that LaCuO_3-x_/meso-Al_2_O_3_ enriched with Cu^3+^ species exhibited significantly higher catalytic activity for NH_3_-SCR than CuO/meso-Al_2_O_3_ counterpart in the low temperature range (100–280 °C), which was attributed to the unique nature of Cu^3+^ species, which had more acid sites and higher redox properties to promote the adsorption and activation of ammonia and NO_x_.

Cu-containing zeolites have been intensively studied for NH_3_-SCR and/or urea-SCR for their applications to stationary and mobile sources [57,77,78]. The NH_3_-SCR performance of Cu-exchanged zeolite catalysts depends on the amount of isolated copper ion sites, SiO_2_/Al_2_O_3_ ratios, and topological structures. The isolated Cu^2+^ species serve as redox sources, the zeolite supplies acidic sites, and the channel structure affects the diffusion of reactants, intermediates, and products. A variety of zeolite catalysts for Fe or Cu ion exchange have been developed, all of which exhibit a wide temperature window, including ZSM-5, SAPO-18, SAPO-34, SSZ-13, SSZ-16, SSZ-39, AFX, BEA, ERI, KFI, LTA, Nu-3, PST-7, RHO, RTH, Sigma-1, UZM-35, etc. [78,79,80,81,82,83]. In terms of the hydrothermal stability, ion-exchanged small-pore zeolites such as Cu-SSZ-13, Cu-SSZ 16, Cu-SSZ-39, Cu-SAPO-18, Cu-SAPO-34, and Cu-KFI are superior to the medium-pore ZSM-5 zeolite catalysts, and finally, Cu-SSZ-13 catalysts have been commercialized for NO_x_ control in diesel-powered vehicles owing to their outstanding NO_x_ removal efficiency and hydrothermal stability [77,79,84]. A comparison of the NH_3_-SCR activity of Cu- and Fe-zeolites showed that at low temperatures (below 350 °C), the former was more active than the latter, but above 350 °C, the latter was more active than the former, which was ascribed to the fact that Cu-zeolites had higher adsorption sites for NH_3_ than Fe-zeolites [85].

**Figure 2:**
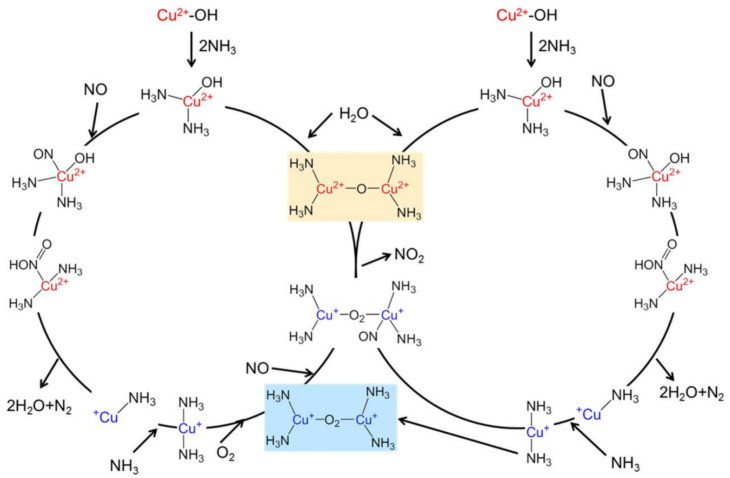
Complete redox cycling mechanism for low-temperature standard NH_3_-SCR derived from DFT calculations that involves two Cu(I) centers in the initiation of the oxidation half-cycle [87]. Adapted from permission from [86]. Copyright 2017, American Chemical Society.

The reaction mechanism over Cu-SSZ-13 is shown in Figure 2 [86]. A mobile NH_3_-complex, monomeric Cu ion was identified to be active in the NO_x_ reduction cycle in the redox mechanism. The Cu^+^ ion complexes thus formed subsequently migrate between the zeolite cages to form dimeric species that are important for O_2_ activation, which is the rate-limiting step essential for the re-oxidation of Cu^+^ to complete the catalytic cycle [87]. Density functional theory (DFT) calculations on a reaction mechanism for low-temperature NH_3_-SCR over Cu-CHA reveal that ammonia-solvated Cu cations, Cu(NH_3_)_2_^+^, are responsible for O_2_ activation as well as the formation of the key intermediates HO-NO and H_2_N-NO and that Brønsted acid site is related to decomposition of HO-NO and H_2_N-NO to N_2_ and H_2_O [88]. Oxygen activation requires pairs of Cu(NH_3_)_2_^+^ complexes, but HO-NO and H_3_N-NO coupling may occur on single complexes [88].

Catalyst deactivation in the presence of SO_x_ in the flue gas can be classified into two categories. One is irreversible deactivation due to the formation of inactive Cu sulfate species through interaction between SO_x_ and active Cu species [89,90,91,92,93,94,95]. The other is reversible deactivation resulting from the deposition of sulfate species (e.g., ABS and AS) [96]. SO_2_ poisoning inhibits the oxidation of NO to NO_2_, resulting in a lower NH_3_-SCR activity below 350 °C [79]. Above this temperature, sulfate compounds are unstable so that the available Cu sites can be sufficient for NH_3_-SCR activity [79]. Therefore, sulfur poisoning beyond 350 °C is insignificant. Table 2 summarizes the effects of H_2_O and SO_2_ on the NH_3_-SCR over some Cu-based catalysts [97,98,99,100,101,102,103,104,105,106,107,108,109]. Among them, CuO@Cu-metal organic frameworks (MOFs) core-shell catalyst [101] and Cu-doped phosphomolybdic acid catalyst [106] showed a relatively stable catalytic performance even in the presence of H_2_O and SO_2_ at low temperatures.

Hammershøi et al. [89] reported that the NH_3_-SCR activity over Cu-SSZ-13 catalysts was significantly inhibited at 160–350 °C after exposure to SO_2_, but preserved above 350 °C. Jangjou et al. [110] compared SO_2_-induced catalyst deactivation at [Cu^II^OH]^+^ and Cu^2+^ sites in Cu-SSZ-13. For Cu^2+^ sites, the NH_3_-SCR activity was inhibited due to the formation of ammonium sulfate, which could be fully recovered by regeneration at >380 °C. This implies that Cu^2+^ sites do not adsorb sulfur during the exposure to SO_2_ [110]. On the other hand, [Cu^II^OH]^+^ sites could adsorb sulfur directly to form Cu bisulfite, causing the irreversible deactivation [110]. Therefore, the sulfur poisoning of [Cu^II^OH]^+^ was much more severe than that of Cu^2+^. H_2_SO_4_ formed in the presence of SO_2_ and H_2_O plays an important role in the formation of ammonium sulfate [111,112]. Another route to the formation of Cu bisulfate is the interaction between SO_2_ and CuO_x_ nanoclusters [113].

The effect of SO_2_ on Cu-SAPO-34 deactivation was also investigated [91,114]. The NH_3_-SCR activity of sulfur-poisoned Cu-SAPO-34 was decreased significantly at 100–500 °C, though sulfur accumulation and zeolite structure collapse were not observed [114]. The deactivation was attributed to the decreased amount of isolated Cu^2+^ ions [91,114]. It was speculated that sulfur poisoning could hinder the copper redox transformation (between Cu^II^ and Cu^I^) in Cu-SAPO-34, which led to the sulfation of Cu sites [115]. In addition, Cu-SAPO-34 favored the formation of stable Al_2_(SO_4_)_3_ species compared with Cu-SSZ-13, resulting in an irrecoverable loss in NH_3_-SCR activity [111].

When screening Cu-containing zeolite catalysts, hydrothermal stability has become an important selection criterion in addition to high NO_x_ conversion over a wide range of reaction temperatures for application in transport vehicles, but this is not as important for low-temperature NH_3_-SCR catalysts for application in stationary sources. Rather, resistance to water and SO_2_ at low temperatures is an additional factor to be considered in the development of NH_3_-SCR catalysts. In order to improve the low-temperature NH_3_-SCR activity of Cu-containing zeolite catalysts, the Cu active sites can be modified by introducing the heteroatoms to facilitate the redox property of Cu and metal oxides to accelerate NO oxidation for ‘Fast NH_3_-SCR’ [116]. Other zeolites with different structures, including AEI and LTA, the small-pore intergrown zeolites, including AFX/CHA and CHA/AEI, and the zeolite morphology can be further examined even though they were excluded because of their poor hydrothermal stability at high temperatures [116]. Additional doping of Y [63] and lanthanides [59,60,61,62,63,64] to Cu-zeolites was reported to be effective for NH_3_-SCR. The composite catalysts such as MnO_2_-CeO_2_/Cu/SSZ-13 showed excellent NH_3_-SCR activity and N_2_ selectivity in the temperature ranges of 125 to 450 °C because more active monodentate nitrate was formed on the surface of the composite catalyst compared with Cu/SSZ-13 alone [99].

Chen et al. [107] synthesized a core–shell structure Cu–Ce–La/SSZ-13@ZSM-5 catalyst by a self-assembly method and applied it to NH_3_-SCR. They observed that Cu–Ce–La/SSZ-13@ZSM-5 with an appropriate shell thickness presented better NH_3_-SCR activity and hydrothermal stability than Cu–Ce–La/SSZ-13 because some metal ions were transferred and redistributed during the assembly of the ZSM-5 shell, resulting in the conversion of [Cu(OH)]^+^–Z to Cu^2+^–2Z species and the functionalization of the shell phase, which was beneficial for the adsorption and activation of NH_3_. Chen et al. [109] synthesized a multifunctional core-shell catalyst with Cu-SSZ-13 as the core phase and Ce-MnO_x_ supported mesoporous silica as the shell phase via self-assembly and impregnation. The core-shell catalyst exhibited excellent low-temperature activity, SO_2_ tolerance, and hydrothermal stability compared with the Cu-SSZ-13 [109]. The Ce-MnO_x_ species dispersed in the shell can rapidly activate NO and oxidize it to NO_2_, which allows the NH_3_-SCR reaction on the core-shell catalyst to be initiated in the shell phase [109]. Meanwhile, Ce-MnO_x_ species can react preferentially with SO_2_ as sacrifice components, effectively avoiding the sulfur inactivation of the copper active sites [109]. This catalyst showed relatively stable NO conversion in the presence of 10% H_2_O and 100 ppm SO_2_ at 250 °C [109]. It is noteworthy that few low-temperature Cu-based catalysts with high resistance to H_2_O and SO_2_ poisoning have been reported.

### 2.2. Fe-Based Catalysts

Various Fe-based catalysts, including single iron oxides, Fe-containing mixed metal oxides, supported iron oxides, supported Fe-containing multicomponent metal oxides, and Fe-containing zeolites, have been reported for NH_3_-SCR due to their excellent redox properties and low cost. Another advantage of Fe-based catalysts is their relatively good resistance to water and SO_2_, although this has only been reported at temperatures above 200 °C [11].

**Figure 3:**
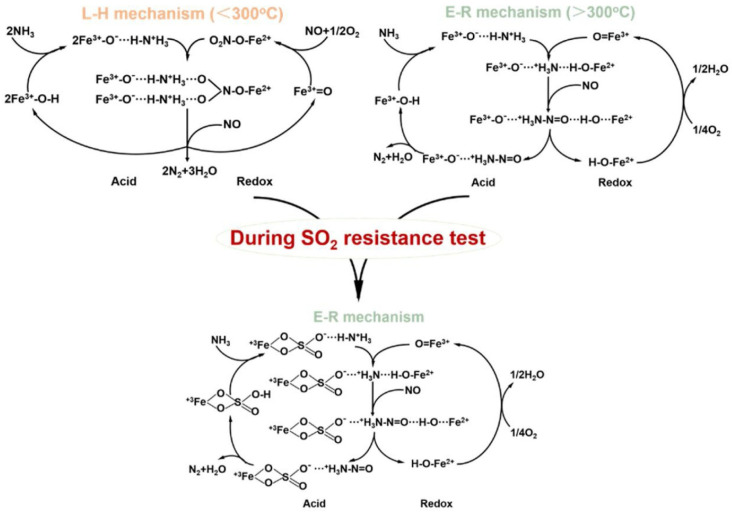
The proposed reaction mechanism before and during the SO_2_ resistance test on the γ-Fe_2_O_3_ catalyst [117]. Adapted from permission from ref [117]. Copyright 2021 Elsevier.

γ-Fe_2_O_3_ has been mainly reported for NH_3_-SCR among various single-phase Fe_2_O_3_ with different crystalline structures (e.g., α, β, γ, and ε) [11]. Yu et al. [117] proposed two different reaction mechanisms over γ-Fe_2_O_3_ (Figure 3). One is the Langmuir–Hinshelwood (L–H) mechanism in which NH_3_ and NO are adsorbed on the active sites, Fe^3+^-OH and Fe^3+^=O, respectively, and reacted to form N_2_ and H_2_O, which is prevalent at temperatures below 300 °C [117]. The other is the Eley–Rideal (E–R) mechanism in which NH_3_ is first chemisorbed on Fe^3+^-OH to form Fe^3+^-O^−⋯^H-N^+^H_3_, which can be further reacted with gaseous NO to produce N_2_ and H_2_O, which is the main pathway at high temperatures above 300 °C [117]. In any case, this reaction requires the cooperation of acidic sites and redox properties. They also confirmed that the NH_3_-SCR activity decreased firstly and then increased slowly after the introduction of SO_2_ at temperatures ranging from 225 to 275 °C [117]. This was ascribed to the formation of iron sulfate species inhibiting the adsorption of NO_x_, thus interfering with the L–H reaction pathway [117]. On the other hand, the iron sulfate species formed enhanced the surface acidity, which promoted the E–R reaction pathway and further promoted NH_3_-SCR activity [117].

Recent work on single Fe oxides has focused on how to increase the low-temperature NH_3_-SCR activity. Qin et al. [118] prepared various Fe_3_O_4_ nanostructures exposed with different crystal planes from MIL-100(Fe) as the Fe precursor and found that the catalysts with more (1 1 1) had better NH_3_-SCR performance than those with (1 0 0) exposure, which they attributed to the preferential exposure of the Fe_3_O_4_ (1 1 1) crystal faces leading to higher adsorbed oxygen concentration and surface acidity. Yang et al. [119] found through DFT calculations that the ‘Fast NH_3_-SCR’ reaction was the dominant pathway for the NH_3_-SCR reaction on Fe_2_-N_6_ catalysts, with the energy barrier of the rate-determining step (HONO formation) being 1.00 eV, much lower than that of other NH_3_-SCR catalysts, enabling excellent low-temperature activity in the temperature window of 300–500 K. Zhang et al. [120] prepared a highly defective α-Fe_2_O_3_ with enhanced acid (Lewis and Brønsted) and redox properties on homoatomic dinuclear sites comprising more positively charged Fe^3+^ and oxygen vacancy-coupled Fe^2+^ ions. The catalyst showed enhanced NH_3_-SCR activity at low temperatures without the addition of other acid transition metals and showed resistance to poisoning of H_2_O and SO_2_ due to the large amount of Brønsted acid [120].

Various metal oxide-promoted iron oxides have been investigated to enhance the cooperation of surface acidity and redox property to facilitate the low-temperature NH_3_-SCR. The positive effects of Mn [121], Nb [122], Mo [123], Ce [124], Sm [125], and W [123,126,127,128] in promoted iron oxides on the catalytic activity have been reported. Table 3 summarizes the effects of H_2_O and SO_2_ on the NH_3_-SCR over some Fe-based catalysts [120,121,124,125,128,129,130,131,132,133,134,135,136,137]. Among them, a relatively stable catalytic performance was observed over Mn-Fe oxides [121] even in the presence of H_2_O and SO_2_ at low temperatures.

It is noteworthy that, except for catalysts containing additional Mn, few Fe-based catalysts are active for NH_3_-SCR at low temperatures (<200 °C) while being resistant to H_2_O and SO_2_ poisoning. Jiang et al. [138] prepared a phosphotungstic acid (HPW)-promoted Fe-based catalyst from MIL-100(Fe) as the Fe precursor by hydrothermal method and reported that active oxygen species-rich γ-Fe_2_O_3_ was the main Fe phase, which enhanced NO adsorption and activation, leading to faster NH_3_-SCR. The role of HPW was to increase the total acidic sites along with promoting the reactivity of NH_3_ adsorbed on Lewis acidic sites at low temperatures. Notably, WO_3_-promoted Fe_2_O_3_ was reported to have a wide temperature window and excellent water and sulfur resistance, showing relatively stable NO conversion in the presence of 100 ppm SO_2_ at 300 °C [128]. The roles of WO_3_ are known to inhibit the crystallization of the Fe_2_O_3_ phase and formation of inactive nitrate, instead increasing Lewis acid sites and appropriate redox properties [127,128]. A kind of composite catalyst, HPW-decorated ring-like Fe_2_O_3_ synthesized via mechanical-chemistry grinding of HPW and Fe_2_O_3_ nanorings prepared by a microwave-assisted hydrothermal method, showed good NH_3_-SCR performance over a wide temperature window of 250–500 °C [139] and an outstanding resistance against SO_2_, showing relatively stable NO conversion in the presence of 10% H_2_O and 200 ppm SO_2_ at 280 °C [132]. Sun et al. [125] reported that Sm modification could weaken the sulfation of active Fe sites in Sm-doped Fe_2_O_3_, which was also supported by the DFT calculation results that SO_2_ could be more easily adsorbed on the Sm/Fe_2_O_3_ catalyst with the adsorption sites located at the Sm atom and its neighboring Fe atoms. They observed that more reactive nitrate species were formed on the sulfated Sm/Fe_2_O_3_ catalyst due to the presence of more un-sulfated Fe sites, which they explained as making the Sm/Fe_2_O_3_ catalyst resistant to SO_2_ poisoning, showing relatively stable NO conversion in the presence of 5% H_2_O and 100 ppm SO_2_ at 275 °C [125]. Tan et al. [140] reported that the low-temperature (<250 °C) NH_3_-SCR activity of FeTiO_x_ catalyst could be dramatically enhanced by CeO_2_ doping, which can be attributed to the presence of a unique Ce-O-Fe structure that contributes to the improvement of redox properties. Chen et al. [124] prepared a single-atom Ce-modified α-Fe_2_O_3_ catalyst by a citric acid-assisted sol–gel method and reported that a high NO conversion was maintained in the presence of 5% H_2_O and 200 ppm SO_2_ at 250 °C. They claimed that the atomic dispersion of the Ce species to maximize the amounts of Fe–O–Ce sites in the Ce-doped FeO_x_ catalyst was critical because the formation of oxygen vacancies in the Fe–O–Ce sites, which could promote the oxidation of NO to NO_2_ and decomposition of ABS, was more favorable than that in the Fe–O–Fe sites in the Ce-free α-Fe_2_O_3_ catalyst [124]. Ma et al. [141] compared a serial of Cu_0.02_Fe_0.2_Ce_y_Ti_1-y_O_x_ catalysts prepared by the sol–gel method and found that Cu_0.02_Fe_0.2_Ce_0.2_Ti_0.8_O_x_ exhibited superior low-temperature NH_3_-SCR performance in the presence and absence of water, which they attributed to the optimal distribution of surface acidity, enhanced surface oxygen content, and surface redox cycle (Ce^4+^ + Fe^2+^ → Ce^3+^ + Fe^3+^). Yao et al. [133] prepared Mn-W-Sb modified siderite catalysts by impregnation method and found that the Mn-doping enhanced adsorbed NO_2_ formation by synergistic catalysis with Fe^3+^ and that the addition of Sb inhibited sulfate formation on the surface of the catalyst in the presence of SO_2_ and H_2_O, showing relatively stable NO conversion in the presence of 5% H_2_O and 100 ppm SO_2_ at 210 °C. Xu et al. [121] prepared various MnFeO_x_ catalysts with different molar ratios and observed high low-temperature NH_3_-SCR activity. The Mn–Fe-0.2 (Mn/Fe = 0.2) catalyst presented excellent SO_2_/H_2_O tolerance, showing a rather stable NO_x_ conversion even in the presence of 5% H_2_O and 50 ppm SO_2_ at 100 °C [121]. Doping Mn not only inhibited the phase transformation of iron oxide (Fe_2_O_3_) but also strengthened the interaction between MnO_x_ and Fe_2_O_3_ due to the electron transfer between them, which led to the formation of Mn–O–Fe [121]. Bai et al. [142] prepared an amorphous metal oxide (FeO_x_-Mn_0.1_O*_y_*) with a large surface area, sufficient oxygen vacancies, and excellent redox properties and observed high adsorption and activation capacities for O_2_ and NO, which further enhanced the catalytic activity at low temperatures (90–240 °C). The incorporation of Mn into the FeO_x_ species suppressed the crystallization of hematite, further increasing the surface area and surface acid sites [142]. In addition, the incorporation of manganese increased the number of oxygen vacancies, which decreased the apparent activation energy of hematite and enhanced the redox properties of the amorphous FeO_x_-MnO_y_ catalyst [142].

The mesoporous Fe-doped CeO_2_ catalyst after modifying organic sulfate functional groups showed excellent activity in a temperature range of 250–450 °C, which was ascribed to the strong electron interaction between Fe^3+^-O-Ce^4+^ species and sulfate groups, which modifies the acidity and redox properties of the catalyst [143]. Wang et al. [144] prepared a series of sulfated modified Fe–Ce composite oxide Fe_1–x_Ce_x_O_δ_-S catalysts and reported that the Fe_0.79_Ce_0.21_O_δ_-S catalyst achieved the low-temperature NH_3_-SCR activity at temperatures of 175–375 °C. They claimed that sulfation formed a large amount of sulfate on the catalyst surface and provided abundant Brønsted acid sites, which enhances NH_3_ adsorption capacity and improves overall NO_x_ conversion efficiency [144]. The introduction of Ce was the main determinant to control the low-temperature activity of the catalyst by modulating its redox ability, and they found that there was a strong interaction between Fe and Ce in the Fe_0.79_Ce_0.21_O_δ_-S catalyst, which changed the electron density around the Fe ions, which weakened the strength of the Fe–O bond and improved the lattice oxygen mobility of the catalyst [144]. In addition, during the reaction, the Fe-Ce composite oxide catalyst showed higher surface lattice oxygen activity and a faster bulk lattice oxygen replenishment rate [144].

The support of the supported iron oxide can increase the dispersion of the iron oxide, providing high surface active sites per mass of iron oxide, provide additional surface acid sites on the support itself and at the interface between the support and the iron oxide, and improve the redox properties of the iron oxide. Different crystal planes of the support (e.g., TiO_2_ [145] and CeO_2_ [146]) of supported iron oxides were compared. Monolayer Fe_2_O_3_ supported on TiO_2_ nanosheets exhibited a better low-temperature NH_3_-SCR activity than that supported on TiO_2_ nanospindles because the former had more acidic sites, oxygen defects, and reactive oxygen species [145]. The iron oxides supported on CeO_2_ nanorods achieved higher catalytic activity for NH_3_-SCR than those supported on CeO_2_ nanopolyhedra, which was explained by the DFT calculation results that the Fe_2_O_3_/CeO_2_ {110} catalyst was more reactive to NO and NH_3_ than the Fe_2_O_3_/CeO_2_ {110} [146]. The Fe_2_O_3_{1 1 3}-TiO_2_ exhibited superior NO_x_ removal capacity and a broader temperature operating range than Fe_2_O_3_{0 1 2}-TiO_2_ and Fe_2_O_3_{0 1 4}-TiO_2_, which can be attributed to the improved redox properties, as well as the presence of additional active oxygen species, surface acid sites, and adsorbed nitrate species on the Fe_2_O_3_{1 1 3}-TiO_2_ catalyst [147].

### 2.3. Mn-Based Catalysts

Mn-based catalysts, including MnO_x_, Mn-containing mixed metal oxides, supported MnO_x_, and supported Mn-containing multicomponent metal oxides, have been regarded as the promising low-temperature NH_3_-SCR catalysts [30,148,149,150]. A comparison of the low-temperature NH_3_-SCR activity between various transition metal oxides supported on TiO_2_ showed that it decreased in the following order: Mn > Cu ≥ Cr >> Co. > Fe >> V >> Ni [151]. First-principles calculations also revealed that the superior oxidative dehydrogenation performance of Mn-based catalysts to NH_3_ lowered the energy barrier for the activation of NH_3_ and reduced the formation of the key intermediate NH_2_NO, the rate-determining step in NH_3_-SCR, over Mn-, Fe-, and Ce-based oxide catalysts [152].

Among various single-phase manganese oxides, including MnO_2_, Mn_5_O_8,_ Mn_2_O_3_, Mn_3_O_4_, and MnO, MnO_2_, and Mn_2_O_3_ were reported to have the highest low-temperature activity per unit surface area and N_2_ selectivity, respectively [153]. The crystallinity and valence state of MnO_x_ affected by the preparation method and pretreatment conditions have been reported to influence the NH_3_-SCR performance [154]. Higher low-temperature NH_3_-SCR activity was obtained for the less crystalline MnO_x_ [155,156]. A comparison of β-MnO_2_ and α-Mn_2_O_3_ showed that the former had a higher NO_x_ conversion and N_2_O production rate per unit surface area than the latter, which was explained by the lower Mn–O bond energy of β-MnO_2_, which promoted the activation of NH_3_ [157]. The birnessite-type MnO_2_ catalyst, prepared from Mn(NO_3_)_2_·4H_2_O, having the fewest OH groups, was reported to exhibit the best catalytic activity and excellent SO_2_ resistance among the same-type catalysts prepared from different Mn precursors [158].

A novel synthetic method was applied to prepare high-surface-area manganese oxide with low crystallinity for this reaction. Mesoporous α-MnO_2_ nanosheets prepared by a solvent-free synthetic method had a high surface area and a mesopore size of 4 nm with large oxygen vacancies and showed 100% NO_x_ conversion under a gas hourly space velocity (GHSV) of 700,000 h^−^^1^ at 100 °C [159]. Xu et al. [160] prepared MnO_x_ catalysts with 3D structure by the hard-template method using KIT-6 as a template possessing high reducibility with abundant surface oxygen species and Mn^4+^ species and reported that more Lewis acid sites and Brønsted acid sites on the surface were beneficial for the adsorption and activation of NH_3_, leading to the higher NH_3_-SCR activity. Chen et al. [161] synthesized a novel MnO_x_ catalyst with a large surface area, small particle size, and more crystalline defects from MOF-Mn_3_(BTC)_2_(H_2_O)_6_ using different amounts of polyvinyl pyrrolidone (PVP) and found that this catalyst had abundant acid sites, Mn^4+^, and surface chemical oxygen, promoting the NH_3_-SCR reaction. Moreover, the high SO_2_ tolerance was also observed because an irreversible sulfurization rate was reduced and an adsorption of active bidentate nitrates and NH_4_^+^ was promoted even in the coexistence of sulfates, showing relatively stable NO conversion in the presence of 50 ppm SO_2_ at 175 °C. Zhang et al. [162] also prepared various MnO_x_ catalysts from MOF-74 under different pretreatment conditions and reported that their redox capability could be controlled by changing the ratio of Mn^4+^/Mn^n+^ and O_α_/(O_α_ + O_β_), resulting in the promotion of the adsorption and oxidation of NO to facilitate the ‘Fast NH_3_-SCR’ reaction. Zhou et al. [163] prepared hydrothermally stable MnO_x_/Al_2_O_3_ catalysts with highly dispersed low-coordinated Mn active sites that were originally created with triethanolamine and observed excellent low-temperature NH_3_-SCR activity because of active low-coordinated Mn species possessing reactive redox sites and Lewis acid sites.

**Figure 4:**
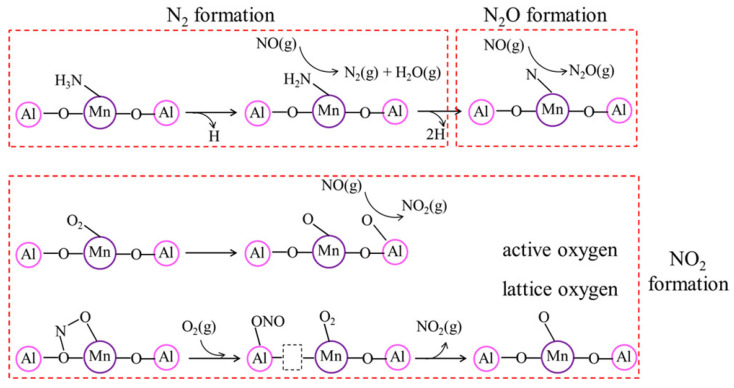
The reaction mechanism of NH_3_-SCR on the Mn/γ-Al_2_O_3_ catalyst calculated by Li et al. [164] Reproduced from ref [164]. Copyright 2019 American Chemical Society.

Li et al. [164] employed DFT calculations for the reaction mechanism of NH_3_-SCR over Mn/γ-Al_2_O_3_ catalyst. NH_3_ is mainly adsorbed on the Lewis acid sites and forms coordinated NH_3_. Subsequently, an N–H bond in the adsorbed NH_3_ can be dissociated to form NH_2_*, which can react with the gaseous NO and generate NH_2_NO*. NH_2_NO* was finally decomposed into N_2_* and H_2_O* (Figure 4). On the surface of the Mn/γ-Al_2_O_3_ catalyst, the adsorbed O_2_ was decomposed into the active oxygen atoms, which could oxidize NO into NO_2_. Among three possible routes for N_2_O formation, such as NO decomposition, deep dehydrogenation of NH_3_, and two-step dehydrogenation of NH_2_NO, the deep dehydrogenation of NH_3_ appears to be mainly responsible over the Mn/γ-Al_2_O_3_ catalyst in both the L–H and E–R reaction mechanisms.

**Figure 5:**
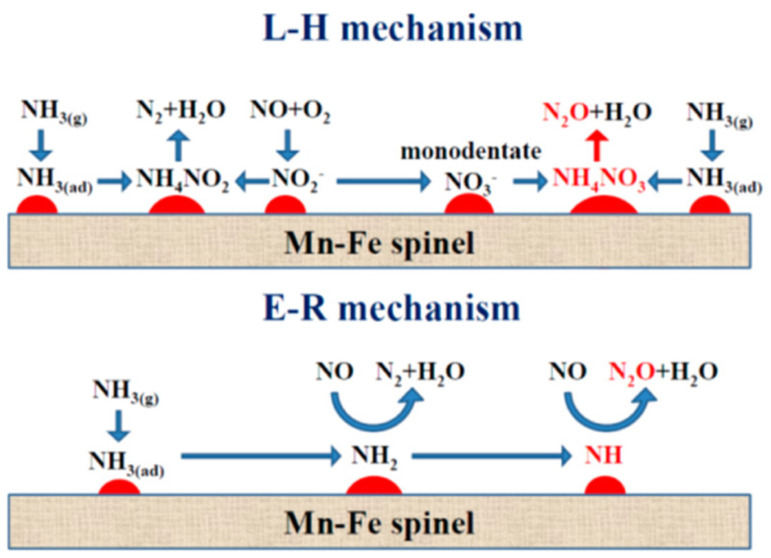
Proposed N_2_O formation mechanisms over Mn–Fe spinel. This schematic diagram is reprinted with permission from ref [166]. Copyright 2014 American Chemical Society.

The formation of N_2_O over Mn-based catalysts was systematically examined over MnO_x_-TiO_2_ catalysts with different Mn/Ti ratios [165]. As the Mn/Ti ratio increased, the MnO_x_ species layer expanded on TiO_2_ support, leading to an increase of redox sites but a decrease of surface acid sites, contributing to the non-selective oxidation of NH_3_ on MnO_x_ species and the over-activation of NH_3_ at the Mn-Ti interface and resulting in the formation of N_2_O. Yang et al. [166] proposed the scheme for N_2_O formation according to the L–H and E–R mechanisms, in which monodentate –NO_3_^−^ (and NH_4_NO_3_*) and –NH are key intermediates for N_2_O formation, respectively (Figure 5). DFT calculations and thermodynamic/kinetic analysis of NH_3_-SCR over α-MnO_2_ with specific (100), (110) and (310) exposure planes showed that the α-MnO_2_ catalyst exposed with the (310) plane exhibited the best NH_3_-SCR catalytic performance and the highest N_2_ selectivity due to the low energy barrier of NH_3_ dehydrogenation and NH_2_NO generation and the difficulty of NH_2_ dissociation [167].

Although manganese oxides exhibit high NH_3_-SCR activity at low temperatures, their application in practical processes is limited due to their sensitivity to SO_2_ and low N_2_ selectivity [21,168]. Therefore, Mn-containing mixed metal oxides and supported Mn-containing metal oxides have been adopted to address these issues. For Mn-containing mixed metal oxides, various second metals including Al [169], Si [170], Ti [171], V [172], Cr [173], Fe [174,175,176,177,178], Co [179,180,181,182], Ni [183], Cu [184,185], Y [186], Zr [187], Nb [188], Mo [189,190,191], Ag [192,193], Sn [194], Sb [195], La [196,197], Ce [197,198,199,200,201,202,203,204], Pr [205], Nd [206], Sm [207,208,209], Eu [210], Gd [211], Dy [212,213], Ho [214], Er [215], Tm [216], Ta [217], W [218,219], and Bi [220] have been examined. The effects of H_2_O and SO_2_ on the NH_3_-SCR activity over unsupported Mn-based catalysts, supported Mn-based catalysts, and core-shell Mn-based catalysts are summarized in Table 4 [161,173,194,220,221,222,223,224,225,226,227,228,229,230,231,232,233,234,235,236,237,238,239,240,241,242,243,244,245,246,247,248,249,250,251,252,253,254,255,256,257,258,259,260,261,262], Table 5 [171,187,189,195,212,215,263,264,265,266,267,268,269,270,271,272,273,274,275,276,277,278,279,280,281,282,283,284,285,286,287,288,289,290,291,292], and Table 6 [293,294,295,296,297,298,299,300,301,302,303,304,305,306,307,308], respectively. Among them, a relatively stable catalytic performance was observed over unsupported Mn-based oxides such as Mn oxides [161], Mn-Cr oxides [173,223], Mn-Fe oxides [224], Mn-Fe-Co oxides [225], Mn-Fe-Mg oxides [226], Mn-Fe-Al oxides [227], Mn-Co oxides [230,232,233,234], Mn-Co-Ce oxides [235], Mn-Co-V oxides [236], Mn-Ni oxides [235,238,239], Mn-Ni-Al oxides [240], Mn-Ni-Fe oxides [244], Mn-Ni-Ce oxides [235], Mn-Zr-Ti oxides [246], Mn-Ce oxides [248,249], Mn-Ce-Ti oxides [250,251,252], Mn-Ce-Sn oxides [248], Mn-Sm-Ti oxides [255,256], Mn-Sm-Fe oxides [257], Mn-Sm-Zr-Ti oxides [258], Mn-Sm-Ce-Ti oxides [259], Mn-Nd oxides [206], Mn-Gd oxides [261], Mn-W-Ce oxides [262], and Bi-Mn oxides [220], and supported Mn-based catalysts such as Mn/Fe-Ti spinel [263], Mn/CeO_2_-ZrO_2_ [266], Mn/CeO_2_-ZrO_2_-Al_2_O_3_ [267], TiO_2_-MnO_x_/CeO_2_-ZrO_2_ [171], Fe-Mn/TiO_2_ [270], Zr-Mn/attapulgite [273], Ce-Mn-V-W/TiO_2_ [277], Nd-Mn/TiO_2_ [278], Ho-Fe-Mn/TiO_2_ [215], Gd-MnO_x_/ZSM-5 [212], NiMnO_x_/activated coke [283], La-Mn-Fe/activated coke [284], Mn/biochar (BC) [285], Zr-Mn/BC [286], MnCe/Granular activated carbon (AC)-carbon nanotubes (CNTs) [291], and MnO_x_-CeO_2_/graphene [292], and Mn-based core-shell catalysts such as MnO_x_@Eu-CeO_x_ [300], Mn-titanium nanotubes@Ce [303], and Fe_2_O_3_@MnO_x_@carbon nanotube (CNT) [306] even in the presence of H_2_O and SO_2_ at low temperatures.

Liang et al. [221] reported that the MnO_x_-SiO_2_ mixed oxide catalyst synthesized by the co-precipitation method showed high NH_3_-SCR activity with high N_2_ selectivity and sulfur resistance because doping SiO_2_ increased the specific surface area of the catalyst, surface acidity, and NH_3_ adsorption. Doping with SiO_2_ inhibited the adsorption of SO_2_ for surface deposition of sulfate, effectively protecting the redox properties of the catalyst so that the MnO_x_-SiO_2_ catalyst showed relatively stable NO conversion in the presence of 100 ppm SO_2_ at 225 °C [221]. Zhu et al. [222] synthesized mesoporous Mn-TiO_x_ with a three-dimensional structure by a solvent-free self-assembling strategy and observed an outstanding sulfur poisoning tolerance. The hierarchical cross-linked structure with a large specific surface area appeared to be favorable for enhancing the synergy between Mn and Ti, exposing more active sites, and dispersing the poisoning species [222]. Wang et al. [309] synthesized a series of Mn_x_Ce_y_Ti_z_ catalysts with three-dimensionally ordered macroporous (3DOM) structure by a soft template method and reported that after adding Ti, the redox capacity of the 3DOM-Mn_3_Ce_1_Ti_1_ catalyst decreased and the N_2_ selectivity was enhanced due to the strong adsorption of NH_3_ on the acid sites, widening the temperature window. Yao et al. [171] found that the TiO_2_-modified MnO_x_/CeO_2_-ZrO_2_ nanorod exhibited higher catalytic activity than the MnO_x_/CeO_2_-ZrO_2_ nanorod due to the larger amount of oxygen vacancy, the higher ratio of Mn^4+^ among Mn species, the higher proportion of surface adsorbed oxygen species among oxygen species, and the improvement of surface acidity. Furthermore, the TiO_2_ modification appropriately weakened the redox properties of the MnO_x_/CeO_2_-ZrO_2_ nanorods, effectively inhibiting the nonselective oxidation of NH_3_ to N_2_O [171]. Finally, TiO_2_-modified MnO_x_/CeO_2_-ZrO_2_ nanorod exhibited excellent H_2_O + SO_2_ tolerance, showing relatively stable NO conversion in the presence of 5% H_2_O and 100 ppm SO_2_ at 200 °C [171]. Xu et al. [256] reported that over a wide operating temperature range (60–225 °C), Ti-doped Sm–Mn mixed oxide showed a superior NH_3_-SCR performance, especially excellent SO_2_/H_2_O resistance. Stable NO_x_ conversion was achieved even in the presence of 5% H_2_O and 100 ppm SO_2_ at 100 °C [256]. The inclusion of Ti can inhibit MnO_x_ crystallization to increase the high specific surface area and the amount of acid sites, while Sm doping can modulate the redox properties to promote NO oxidation and inhibit NH_3_ nonselective oxidation [256].

Jiang et al. [282] reported that doping V_2_O_5_ to Mn-Ce/AC enhanced the NO conversion, N_2_ selectivity, and SO_2_ tolerance than Mn-Ce/AC because doping with V_2_O_5_ enhanced surface acidity and enriched surface chemisorbed oxygen. The stronger surface acidity of Mn-Ce-V/AC inhibited the competitive adsorption of SO_2_ and suppressed the reaction between adsorbed SO_2_ and NH_3_ species [282]. In addition, the cluster of vanadium oxide species partially prevented the dispersed Mn-Ce solid solution from being sulfated by SO_2_ [282]. This catalyst showed relatively stable NO conversion in the presence of 10% H_2_O and 100 ppm SO_2_ at 200 °C [282]. Li et al. [236] reported that the Mn_0.50_Co_0.49_V_0.01_O_x_ catalyst exhibited superior catalytic performance over a wide temperature window (162–508 °C) among ternary MnCoVO_x_ catalysts and also displayed enhanced SO_2_ tolerance, showing relatively stable NO conversion in the presence of 5% H_2_O and 100 ppm SO_2_ at 200 °C.

Gao et al. [173] prepared Cr-Mn mixed-oxide catalysts by citric acid method and observed good low-temperature NH_3_-SCR performance at 100–225 °C owing to their high specific surface area, more active sites (Mn^3+^ and Mn^4+^), and effective electron transfer (Cr^5+^ + 2Mn^3+^ → Cr^3+^ + 2Mn^4+^). The stronger Mn–O binding energy and weaker dehydrogenation ability in CrMn_2_O_4_ spinel were the main reasons for the lower N_2_O by-product than Mn_3_O_4_ [173]. The CrMn_2_O_4_ spinel showed relatively stable NO conversion in the presence of 10% H_2_O and 150 ppm SO_2_ at 200 °C [173]. The formation of Cr(III) sulfate could protect Mn active sites from sulfating. Furthermore, the transformation of -HSO_3_ and SO_4_^2−^ to (H⋯SO_4_^2−^) can provide new Brønsted acid sites for ionic NH_4_^+^, enhancing the NH_3_-SCR activity via ‘Fast-NH_3_-SCR’ (NO_2_ + NH_4_^+^ → NH_4_NO_2_ → N_2_ + 2H_2_O) [173].

The DFT calculation results indicated that Fe doping promoted the adsorption of NH_3_ on the Mn sites over the β-MnO_2_ (110) surface, weakened SO_2_ adsorption capacity, and limited the oxidation of SO_2_, which promoted the decomposition of ammonium sulfate and relieved the catalytic site blockage on the catalyst surface [310]. DFT calculation demonstrated that the thermodynamically most favored structure after Fe doping was MnO_2-x_, and the structure remained stable as the doping amount increased [311]. The adsorption behavior of gas molecules on MnO_2-x_ showed that NH_3_ and H_2_O preferred to adsorb on the Mn Lewis acid sites, while NO and SO_2_ preferred to adsorb on the O sites. MnO_2-x_ with enhanced acidity after Fe doping showed good SO_2_ tolerance [311]. Wu et al. [312] prepared various Mn/TiO_2_ catalysts promoted with different transition metals, including Fe, Cu, Ni, and Cr, by the sol–gel method and found that more NO could be oxidized to NO_2_ and nitrate and then reacted with NH_3_, thus leading to the enhanced NH_3_-SCR activity at low temperatures. The promoting effect of Fe on Mn-Fe composite oxide catalysts was explained by the fact that Fe promoted NO oxidation and generated more monodentate and bidentate nitrate adsorption species on the catalyst surface [312]. It was also reported that the addition of Fe enhanced the amount and intensity of Brønsted and Lewis acid sites, which promoted the absorption of NH_3_ to form active intermediates, thereby improving the low-temperature NH_3_-SCR performance [313].

Jiang et al. [232] prepared MnO_2_–CoO_x_ catalysts with different Mn/Co ratios using PVP as an assistant to control the morphology and exposed crystal faces of composite oxides. The best Mn–Co oxide showed stable NO_x_ conversions in the presence of 5% H_2_O and 100 ppm SO_2_ at 160 °C [232]. Feng et al. [314] prepared a series of Mn-Co mixed metal oxides, Mn_y_Co_3–y_O_x_, by an ultrasonic technology followed by a hydrothermal treatment and found that they exhibited much better NH_3_-SCR performance than single metal oxides (MnO_x_ or CoO_x_). This can be attributed to the strong interaction between MnO_x_ and CoO_x_, which reduces the crystallinity of the Mn_y_Co_3–y_O_x_ catalyst, increases the specific surface area, establishes the redox cycle of Co^3+^ + Mn^3+^ → Co^2+^ + Mn^4+^, improves the reducibility, enhances the surface acid sites, and accelerates the reaction between adsorbed NO_x_ species and coordinated NH_3_ bound to Lewis acid sites [314]. Chen et al. [233] prepared a MnCoO_x_ catalyst using glucose as the forming pore agent and urea as the structural direction reagent and found that it exhibited higher low-temperature activity and SO_2_ resistance than pure MnO_x_ catalysts. They revealed that the high surface area, abundant chemisorbed oxygen species, and the redox cycle (Co^3+^ + Mn^3+^ → Mn^4+^ + Co^2+^) collectively account for the enhanced catalytic activity of MnCoO_x_ catalysts [233]. The Mn(5)Co(5)O_x_ catalyst showed stable NO_x_ conversions even in the presence of 5% H_2_O and 50 ppm SO_2_ at 125 °C [233]. Zhang et al. [281] prepared a highly active Ce/CoMnAl from layered double hydroxides (LDHs), which showed low-temperature NH_3_-SCR with good thermal stability and SO_2_/H_2_O resistance. The Ce_0.5_/Co_1_Mn_0.5_Al_0.5_O_x_-layered double oxide (LDO) showed relatively stable NO_x_ conversion at 200 °C for 10 h with 100 ppm SO_2_ in the feed stream [281].

Gao et al. [238] reported that the Mn-Ni spinel nanosheet (NiMn_2_O_4_) prepared by the urea hydrolysis hydrothermal synthesis exhibited excellent resistance to H_2_O and SO_2_ in the low temperature range at 150–300 °C. This catalyst showed stable NO_x_ conversions even in the presence of 150 ppm SO_2_ at 175 °C [238]. Ni-doped MnO_x_ catalysts prepared by the solvent-free doping method maintained excellent low temperature (100–200 °C) NH_3_-SCR activity, which was attributed to the fact that the appropriate nickel loading could effectively adjust the surface lattice oxygen activity and acidity of the catalyst, ensuring its ability to activate NH_3_ at low temperatures [315]. Liu et al. [316] reported that NiMn_2_O_4_ catalysts prepared via the solvothermal method exhibited excellent NH_3_-SCR performance in the temperature range of 85 to 285 °C due to their large specific surface area, high Mn^4+^/Mn^n+^ ratio, sufficient O_α_, appropriate acidity, and redox ability. Yan et al. [240] reported that the Ni_1_Mn_0.5_Al_0.5_O_x_ showed the highest NH_3_-SCR activity at 100–250 °C among LDHs-derived NiMnAlO_x_ catalysts because of more active species, abundant surface oxygen, moderate acidic sites, and redox properties. This catalyst also exhibited better resistance to SO_2_ with fewer sulfate species deposited on the surface, showing relatively stable NO conversion in the presence of 5% H_2_O and 100 ppm SO_2_ at 200 °C [240]. Hou et al. [241] prepared a series of highly dispersed Ni_4-x_Mn_x_AlO_y_ catalysts derived from LDHs and reported that the optimal Ni_4-x_Mn_x_AlO_y_ catalyst exhibited high NH_3_-SCR performance at a low temperature range of 120~210 °C but was deactivated in the presence of 5% H_2_O and 100 ppm SO_2_ at 210 °C. Yan et al. [244] prepared NiMnFeO_x_ catalysts by the LDH-derived oxide method and reported that the optimized Ni_0.5_Mn_0.5_Fe_0.5_O_x_ catalyst had the best NH_3_-SCR activity, excellent N_2_ selectivity, a wider active temperature range (100–250 °C), higher thermal stability, and better H_2_O and/or SO_2_ resistance. This catalyst showed relatively stable NO conversion even in the presence of 5% H_2_O and 100 ppm SO_2_ at 200 °C [244]. Wang et al. [283] reported that NiMnO_x_ supported on active coke prepared by deposition-precipitation method showed the high NH_3_-SCR activity because of the higher content of (Mn^3+^ + Mn^4+^), the high ratio of Mn^3+^/Mn^4+^, and Ni^3+^/Ni^2+^. This catalyst showed a stable NO conversion even in the presence of 200 ppm SO_2_ at 200 °C [283].

Doping MnO_x_ and CeMnO_x_ with Y did not improve NO conversion, but it broadened the range of high N_2_ selectivity compared with the undoped case [186]. The addition of Zr to Fe-Mn-Ti oxide catalysts was reported to decrease the deactivation rate in the presence of 100 ppm SO_2_ at 150 °C [187]. Chen et al. [285] prepared several metal (Zr, Ni, and Co) oxide-doped BC-supported Mn oxide (MnO_x_) catalysts by the impregnation method and found that the Zr-Mn/BC catalyst exhibited the best NH_3_-SCR activity among them because of the high concentration of Mn^4+^, more surface oxygen (O_α_), excellent redox property, and more Lewis acid sites and Brønsted acid sites. Furthermore, the Zr-Mn/BC catalyst showed stable NO conversion in the presence of 5 vol% H_2_O and 100 ppm SO_2_ at 200 °C [285]. Che et al. [317] reported that the highly dispersed active MnO_x_ species supported on the amorphous structure of ZrTiO_x_ had a uniquely bridged Mn^3+^ bonded with the support through oxygen linked to Ti^4+^ and Zr^4+^, respectively, which regulated the optimal oxidizability of the MnO_x_ species, leading to high NH_3_-SCR activity and N_2_ selectivity.

Zhou et al. [188] found that doping Nb_2_O_5_ could effectively enhance the NH_3_-SCR performance and N_2_ selectivity of the Zn-Mn-Ce/AC catalyst. Meanwhile, Nb_2_O_5_ could significantly improve the SO_2_ poisoning resistance of the Zn-Mn-Ce/AC catalyst, showing relatively stable NO conversion in the presence of 100 ppm SO_2_ at 225 °C because Nb_2_O_5_ could react with SO_2_ in a preferential way to restrain the sulfuration of manganese and cerium oxides on the catalyst [188]. More importantly, Nb_2_O_5_ reacted with SO_2_ to form Nb sulfate and then formed a new acidic site on the Zn-Mn-Ce/AC catalyst surface, which promoted the adsorption of NH_3_ and inhibited the adsorption of SO_2_, then restricted the reaction between NH_3_ and SO_2_ and hindered the formation of ammonium sulfate channels [188]. The promotional effect of Mo in Mo-MnO_x_ catalyst was reported to be due to its higher Mn^4+^ content, abundant surface adsorbed oxygen (O_α_), and more acid sites [189]_._ Li et al. [318] employed Mo and V to simultaneously fine-tune the acid and redox sites of MnO_x_ and found that the adjusted Mn_0.90_Mo_0.09_V_0.01_O_x_ catalyst demonstrated excellent low-temperature activity and a significantly broader active temperature window.

Chang et al. [194] reported that SnO_2_–MnO_x_–CeO_2_ catalysts prepared by a co-precipitation method showed remarkably high activity, N_2_ selectivity, and SO_2_ resistance at temperatures in the range of 200–500 °C, which can be due to the significant enhancement of Lewis acid sites generated by surface sulfation during the SO_2_-containing NH_3_-SCR reaction. They also explained that the high SO_2_ tolerance can be ascribed to the inhibited formation of MnSO_4_ [194]. This catalyst showed stable NO_x_ conversions in the presence of 12% H_2_O and 100 ppm SO_2_ at 110 °C [194]. Wang et al. [268] synthesized Sn-doped modified CoAl-LDH by a hydrothermal method and used it as a support for the preparation of the active hexagonal sheet structure of the MnO_x_(0.25)/CoSn_3_Al-LDO catalyst, which also showed relatively stable NO conversion at 200 °C after the introduction of 150 ppm SO_2_. Sn doping increased the ratios of Mn^4+^/Mn^3+^ and Co^3+^/Co^2+^ as well as the adsorbed oxygen amount on the catalyst surface, improved the redox capacity of the catalyst, and increased the number of acid sites, resulting in the excellent low-temperature NH_3_-SCR performance [268].

Xie et al. [195] prepared a series of xSb-4Ce-10Mn/TiO_2_ (x = 0, 2, 3, 4, 5, 6) catalysts by a reverse co-precipitation method and observed that the addition of Sb^5+^/Sb^3+^ slightly decreased the catalytic activity but helped improve the H_2_O/SO_2_ resistance. They found that the Sb^5+^/Sb^3+^ addition decreased the Mn^4+^ and Ti^4+^ content but increased the Ce^3+^, surface adsorbed oxygen content, and the surface area, which promoted the decomposition of AS and inhibited the formation of MnSO_4_ [195]. However, stable NOx conversion was not achieved in the presence of 5% H_2_O and 100 ppm SO_2_ at 200 °C [195].

Li et al. [319] reported that the addition of Mn to ceria greatly promotes the formation of surface oxygen vacancies that readily capture O_2_ from the air and form surface reactive oxygen species (superoxide and peroxide), which efficiently oxidize NO to NO_2_ and then promote the ‘Fast NH_3_-SCR’ reaction. Rong et al. [206] prepared the MnXO_x_ catalysts (i.e., MnSmO_x_, MnNdO_x_, and MnCeO_x_) by the reverse co-precipitation method and found that the MnCeO_x_ catalyst showed the best low-temperature catalytic activity and excellent H_2_O + SO_2_ resistance because of the largest amount of acid sites and the best reducibility among these MnXO_x_ catalysts. However, stable NO_x_ conversion was not achieved over these catalysts in the presence of 5% H_2_O and 100 ppm SO_2_ at 175 °C [206]. Fang et al. [320] reported enhanced NH_3_-SCR activity and SO_2_ tolerance of Ce-modified birnessite-MnO_2_ and attributed it to the preferential adsorption and oxidation of SO_2_ onto Ce species to form a new adsorption site for NH_4_^+^, Ce_2_(SO_4_)_3_, which protects the Mn active site from sulfidation and deactivation.

It is generally accepted that homogeneous metal doping into the MnO_x_ matrix is beneficial for NH_3_-SCR activity. However, Cheng et al. [321] claimed that the presence of crystalline Mn_3_O_4_ and its interfacial interaction with CeO_2_ played a significant role in boosting the NH_3_-SCR performance, suggesting the great synergy between CeO_2_ and the crystalline phase of Mn_3_O_4_, which were responsible for NO_x_ adsorption and the formation of NH_2_ species through the activation of NH_3_, respectively. Zhu et al. [322] prepared a series of Ce_x_-Mn-Ti_y_ catalysts by the co-precipitation method and found that the best NH_3_-SCR activity was obtained over the Ce_0.1_-Mn-Ti_0.1_ catalyst with the proper redox ability, abundant acid sites, high content of Mn^4+^ and Ce^3+^, and surface-adsorbed oxygen. Wei et al. [251] prepared Mn–Ce–Ti–O composite aerogels with large mesopore size and reported that the Mn–Ce–Ti–O catalyst calcined at 600 °C showed stable NO conversion in the presence of 5 vol% H_2_O and 100 ppm SO_2_ at 200 °C, which they attributed to its large pore size, averaging 32 nm, and abundant Lewis acid sites, as the former promoted the decomposition of ABS and the latter reduced the vapor pressure of NH_3_. Teng et al. [323] prepared a series of rare-earth (Ce, La, Nd, and Y) oxide-doped Mn/Al_2_O_3_ catalysts by the impregnation method and found that rare earth oxide addition significantly enhanced the NH_3_-SCR catalytic activity of Mn/Al_2_O_3_, with CeMn/Al_2_O_3_ obtaining the highest activity at 125–300 °C. Except for Y_2_O_3_, the rare earth oxide additives promoted the surface distribution of Mn elements and enhanced the ratio of chemisorbed oxygen and Mn^4+^ on the surface of Mn/Al_2_O_3_ [323]. In addition, the introduction of rare earth oxides enhanced the reducibility of MnO_2_ species and obtained a larger amount of weak acid sites [323]. Contrary to some reports on the promotional effect of Ce in Ce-MnO_x_ catalysts, Gevers et al. [324] reported that the intrinsic NH_3_-SCR activity of the Mn active sites is not positively affected by Ce species at low temperatures based on the fact that the surface-area normalized activity did not increase by Ce addition. They concluded that Ce decreased the average oxidation state and activity of Mn active species and was just a structural promotor, increasing catalyst surface area, and that addition of Ce suppressed second-step oxidation reactions and thus N_2_O formation by structurally diluting MnO_x_ [324]. The diffuse reflectance infrared Fourier transform spectroscopy (DRIFTS) analysis showed that N_2_O was mainly generated by the reaction between NO and excessive hydrogen abstraction of NH_3_ (i.e., forming NH species) and that Ca addition inhibited the formation of NH species on the catalyst surface, thereby reducing the formation of N_2_O [325]. Ca addition could also lead to a decreased formation of NO_2_, which could react with NH_3_ to form N_2_O, contributing to the promoted selectivity of N_2_ over Ca-modified Ce-Mn/TiO_2_ [325].

Wu et al. [205] reported that the Pr modification could boost the MnCeO_x_ catalysts’ NH_3_-SCR activity and SO_2_ resistance because of the expanded specific surface area, the improved dispersion of active components, the enhanced surface acidity and redox ability, and the generated more chemisorbed oxygen (O_ads_) species. The MnCePrO_x_-0.3 catalyst exhibited stable NO conversion even in the presence of 5 vol% H_2_O and 100 ppm SO_2_ at 250 °C [205]. Wang et al. [259] reported that the MnCeSmTiO_x_ catalyst preserved higher catalytic performance after introducing H_2_O and SO_2_ compared with the catalysts without adding Sm. They claimed that the synergistic effect of the Lewis acid sites and oxidation catalytic sites of mixed oxides was responsible for NH_3_-SCR by following the L–H mechanism [261]. Doping Sm into MnCeSmTiO_x_ was reported to be able to increase oxygen vacancies and transfer electrons to Mn^4+^ and Ce^4+^, promoting the formation of active adsorbed NO_2_, bidentate nitrate, and bridging nitrate intermediates and suppressing SO_2_ poisoning by inhibiting the oxidation of SO_2_ by Mn^4+^ and Ce^4+^ [259]. The MnCeSmTiO_x_ catalyst exhibited a rather stable NO conversion even in the presence of 5% H_2_O and 200 ppm SO_2_ at 200 °C [259]. Kim et al. [326] reported that the addition of Sm to MnO_x_/TiO_2_ increased the redox properties and the proportion of Mn^4+^ in MnO_x_ catalysts, and the addition of Ce further improved the redox properties of Sm-MnO_x_/TiO_2_ catalysts. They also reported that when exposed to SO_2_/O_2_, Sm and Ce-modified MnO_x_/TiO_2_ not only inhibited the degradation but also increased the N_2_ selectivity by inhibiting the formation of N_2_O_4_ intermediate species, thereby inhibiting the formation of N_2_O [326].

The addition of Eu to MnO_x_/TiO_2_ was reported to be effective against the resistance of SO_2_ (i.e., NO_x_ conversion gradually decreased but reached a steady-state value in the presence of 100 ppm SO_2_ at 150 °C) [279]. Wang et al. [210] prepared a series of MEuMnO_x_ ternary oxides (M = Ce, Ni, Co, Sb, Sn, Mo) by one-pot co-precipitation method and observed that CeEuMnO_x_ ternary oxide catalysts showed a high NH_3_-SCR activity at 100–250 °C and stable NO_x_ conversions in the presence of 10 vol% H_2_O and 50 ppm SO_2_ at 230 °C. The facile electron transfer through the redox cycle of Mn^3+^ + Ce^4+^ → Mn^4+^ + Ce^3+^ and enhanced oxygen mobility can promote the formation of more Mn species in high oxidation states and chemisorbed oxygen, accelerating oxidation of NO, and the adsorbed NO_2_ formed can facilitate the ‘Fast NH_3_-SCR’ reaction to improve low-temperature activity [210]. The addition of Ce to the EuMnO_x_ catalyst boosts adsorption of NH_3_ and NO_x_ species. NH_3_ species are activated as crucial intermediates (NH_2_) to promote the NH_3_-SCR reaction [210]. Guan et al. [211] synthesized a series of Gd-modified MnO_x_/ZSM-5 catalysts via a citric acid–ethanol dispersion method and found that the Gd-modified MnO_x_/ZSM-5 catalyst presented the higher catalytic activity and better SO_2_ resistance than MnO_x_/ZSM-5 in the presence of 100 ppm SO_2_. The high catalytic performance was mainly owing to the large surface area, enriched Mn^4+^ and surface chemisorbed oxygen species, strong redox properties, and the proper acidity of the catalyst [211]. The addition of Gd inhibited the reaction between the SO_2_ and MnO_x_ active sites to form inactive bulk manganese sulfate, resulting in high SO_2_ resistance [211]. The GdMn/Z-0.3 catalyst showed a rather stable NO conversion in the presence of 10% H_2_O and 100 ppm SO_2_ at 180 °C [211]. Gao et al. [212] synthesized Dy-doped MnFe oxides with the morphology of one-dimensional nanowire by using the electrospinning method and found that the doping of Dy with an appropriate amount enhanced the surface Mn^4+^ concentration and surface chemical adsorption of oxygen and increased adsorption capacity for NH_3_ and NO. Zhuang et al. [214] investigated the effect of SO_2_ on the low-temperature NH_3_-SCR activity for Ho-modified Fe-Mn/TiO_2_ catalyst. The Fe_0.3_Ho_0.1_Mn_0.4_/TiO_2_ catalyst showed excellent SO_2_ durability at 120 °C when the concentration of SO_2_ was lower than 400 ppm, and the catalytic activity could recover considerably after the SO_2_ supply was interrupted [214]. When the concentration of SO_2_ was increased to 1000 ppm, the deactivation behavior was irreversible, but the deactivated catalyst could be regenerated after thermal reduction (350 °C) with 5% NH_3_ [214]. Zhao et al. [304] synthesized the Ho-modified titanium nanotubes (TNTs)@MnO_x_, where the manganese oxide active species was confined in a Ho-modified TNTs, and observed the improved SO_2_ resistance and N_2_ selectivity during NH_3_-SCR at low temperatures, showing NO conversion, which decreased slowly over time in the presence of 100 ppm SO_2_ at 180 °C. They claimed that the pore confinement effect of Ho-TNTs on Mn increased the dispersion of Mn, thereby promoting the interfacial effect between Mn and Ho, and the electron synergy between Mn and Ho inhibited the electron transfer between SO_2_ and Mn, thus preventing the poisoning of SO_2_ [302]. They also found that the interaction between Ho and Mn contributed to the proper redox ability to induce electron transfer to inhibit the generation of Mn^4+^, thereby achieving high N_2_ selectivity [302].

Zha et al. [218] found that the addition of tungsten created more Brønsted acid sites and reduced the energy barrier for NO_x_ species adsorbed on the surface. They also claimed that the formed NH_3_ species and NO_x_ species of MnCeW/m-TiO_2_ were more reactive due to the promotional effects of W than those of MnCe/m-TiO_2_ [218]. Liu et al. [327] reported the promotional effect of W-doping on the CoMn_2_O_4_/TiO_2_ catalyst on the resistance to H_2_O at low temperature. The superior performance of the CoMn_2_O_4_/W-TiO_2_ catalyst was ascribed to its unique spinel structure, mesoporous structure, highly dispersed tungsten species, and larger surface acidity [327]. The electron cycle among Mn, Co, W, and Ti helped to keep the Mn^3+^/Mn^4+^ and Co^3+^ at high concentrations and improve lattice oxygen mobility [327]. Both Lewis and Brønsted acid sites were generated by the incorporation of tungsten in the presence of H_2_O, which enhanced NH_3_ adsorption and thus promoted the E–R reaction pathway [327]. In addition, the N_2_ selectivity was significantly enhanced by H_2_O, which could be attributed to the decreased redox activity [327].

**Figure 6:**
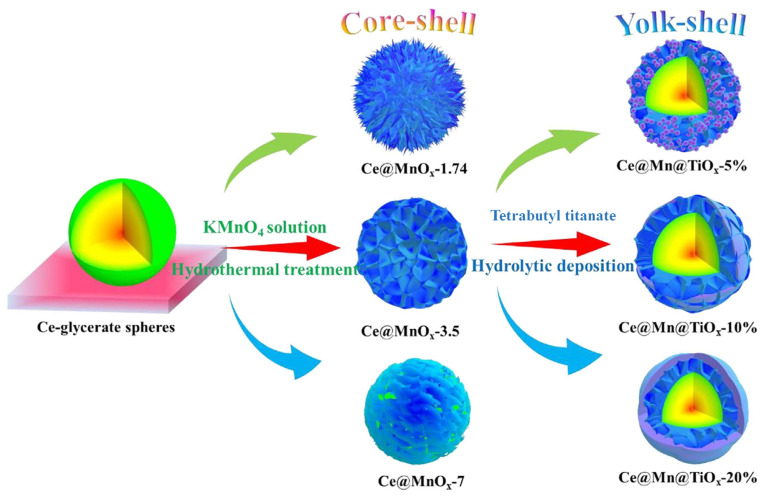
The schematic illustration of the construction for yolk-shell Ce@Mn@TiO_x_ [306]. Adapted from permission from ref [306]. Copyright 2021 Elsevier.

Yan et al. [305] assembled a confined MnCeO_x_ catalyst using a mesoporous zeolite (ZSM-5) as the shell and Mn-Ce oxides as the active core (MnCeO_x_@ ZSM-5) with a simple one-pot method and found that the novel MnCeO_x_@ ZSM-5 catalyst displayed enhanced water and SO_2_ resistance as compared with the MnCeO_x_ supported on ZSM-5 (MnCeO_x_/ZSM-5) and its precursor (MnCeO_x_@Al-SiO_2_), which was ascribed to the zeolite shell’s shielding effect and the synergy between the alumina-silica zeolite shell’s acidic properties and the mixed oxide cores’ redox properties. This catalyst showed relatively stable NO conversion in the presence of 5% H_2_O and 100 ppm SO_2_ at 300 °C [305]. Cai et al. [328] fabricated MnFe@CeO_x_@TiO_x_ nanocage with a yolk-shell structure, where the CeO_2_ shell could effectively increase the oxygen vacancy defect sites and the TiO_2_ shell could remarkably improve the surface acid sites, which exhibited excellent NH_3_-SCR performance in the temperature range of 120–240 °C, which was ascribed to the increased proportion of active species (Mn^4+^, Fe^3+^, Ce^3+^, and O_ads_) and enhanced interaction between metal oxides. Huang et al. [304] prepared a series of yolk-shell-structured Ce@Mn@TiO_x_, where the TiO_2_ shell could provide more surface acid sites to promote the adsorption and activation of ammonia (Figure 6). The Ce@Mn@TiO_x_ exhibited excellent NH_3_-SCR performance at low temperature and H_2_O and SO_2_ tolerance, showing relatively stable NO conversion in the presence of 100 ppm SO_2_ at 200 °C [304]. Qiao et al. [329] prepared various MnO_x_-ZSM-5 catalysts with different MnO_x_ locations and found that MnO_x_ clusters dispersed on the outside of ZSM-5 exhibited higher catalytic activity at low temperature than MnO_x_ nanoparticles encapsulated in the channels of the support because the excellent redox ability and the dominant Mn^3+^ species over the former one improved the oxidation of NO to NO_2_ and the formation of unstable NO_x_ intermediates, enhancing the low-temperature NH_3_-SCR activity. Ran et al. [330] synthesized a series of MnO_x_-CeO_x_ confined in the mesopores of SBA-15 and found that they displayed enhanced resistance to SO_2_. Guo et al. [331] reported that the catalyst with larger mesopores exhibited much improved sulfur resistance. Zhang et al. [307] synthesized a core-shell structural catalyst, carbon nanotube (CNT)-supported MnO_x_ and CeO_x_ nanoparticles coated with mesoporous TiO_2_ sheaths (mesoTiO_2_@MnCe/CNTs), in which the meso–TiO_2_ sheaths could prevent the generation of ammonium/manganese sulfate species from blocking the active sites, resulting in a higher SO_2_-tolerance during NH_3_-SCR. The reversible deactivation was observed over this catalyst due to the presence of 200 ppm SO_2_ at 300 °C [307]. Yan et al. [305] prepared the core-shell MnCeO_x_@mesorporus ZSM-5 catalysts, in which a mesoporous zeolite (ZSM-5) covers the active core Mn-Ce oxides as the shell, and observed enhanced water and SO_2_ resistance during NH_3_-SCR owing to the zeolite shell’s shielding effect, which hinders the formation of sulfate species, and the synergy between the alumina-silica zeolite shell’s acidic properties and the mixed oxide cores’ redox properties. Zhao et al. [303] constructed a Mn-TNTs@Ce catalyst, where MnO_x_ was confined in TNT via in situ introduction following Ce ion exchange into the skeleton of TNTs. The nanotube-confined structure improved the surface acidity to restrain SO_2_ adsorption, and Ce species acted as a protective site protecting Mn from SO_2_ attacking [303]. Furthermore, the short-range structure Ce-O-Ti promoted the electron transformation between Mn and Ce to suppress the formation of -NH and NH_4_NO_3_, resulting in inhibiting the generation of N_2_O [303]. It is remarkably that the Mn-TNTs@Ce catalyst showed a relatively stable NO conversion even in the presence of 5 vol% H_2_O and 100 ppm SO_2_ at 200 °C [303].

It is noteworthy that several promising Mn-based catalysts capable of performing NH_3_-SCR at low temperatures, even in the presence of H_2_O and SO_2_, have been reported; however, most of them have only been shown to be stable for short reaction times, and catalyst analysis of post-reaction samples has been limited. For previously reported promising catalysts, long-term lifetime tests should be performed at low temperatures using flue gases containing H_2_O and SO_2_ to observe deactivation phenomena, and real-time analysis should be performed to identify and validate detailed mechanisms of resistance to H_2_O and SO_2_.

### 2.4. Ce-Based Catalysts

Since cerium-exchanged sodium-type mordenite (CeNa-MOR) was first reported to be active for NH_3_-SCR over a wide temperature range of 250–560 °C [332], unsupported and supported ceria, metal-doped ceria, and multicomponent Ce-containing mixed metal oxides [333,334,335] have been applied to NH_3_-SCR [336]. While NH_3_-SCR activity was reported to be dependent on ceria morphology (i.e., nanosphere is better than nanocube), unsupported ceria showed relatively low catalytic activity at low temperatures, even after sulfation to provide surface acidic sites [337]. Therefore, metal-doped ceria and multicomponent Ce-containing mixed metal oxide catalysts have received much attention for application in low-temperature NH_3_-SCR. Since Cu-, Fe-, and Mn-containing Ce-based catalysts have already been covered in the previous sections, this section mainly introduces Ce-based catalysts with other metals. The effects of H_2_O and SO_2_ on the NH_3_-SCR activity over Ce-based catalysts are summarized in Table 7 [338,339,340,341,342,343,344,345,346,347,348,349,350,351,352,353,354,355,356,357,358,359,360,361,362,363,364,365,366,367,368,369,370,371,372,373,374,375,376]. Among them, a relatively stable catalytic performance was observed over CO-pretreated CeO_2_ [338], F-Ce-Ti oxides [345], P-CeO_2_/TiO_2_ [346], Ti-Sn-Ce-O_x_ [352], and CeBi/TiO_2_ [353] even in the presence of H_2_O and SO_2_ at low temperatures.

Zhou et al. [344] utilized bimetallic MOFs to prepare CeMO_x_ (M=Ti, Cu) catalysts, allowing the homogeneous distribution of promoters. The CeTiO_x_ catalyst with high acidity and good redox properties with abundant Ce^3+^ and Ti^4+^ showed high NH_3_-SCR activity from 180 to 300 °C and maintained stable performance in SO_2_/H_2_O at 225 °C [344]. Meanwhile, the aliovalent substitution of ceria by Cu^2+^ in CeCuO_x_ formed oxygen vacancies and enhanced its redox properties but led to poor N_2_ selectivity due to NH_3_ over-oxidation [344]. Guo et al. [353] used Bi as the modifier to boost the performance of the Ce/TiO_2_ catalyst for NH_3_-SCR. The CeBi/TiO_2_ catalyst with a proper Bi content showed a rather stable NO conversion in the presence of 5% H_2_O and 100 ppm SO_2_ at 150 °C because the addition of Bi could generate more Ce^3+^ and chemisorbed surface oxygen species, along with enhanced redox capability and surface acidity [353].

Jin et al. [371] investigated the role of each metal component in Ce-Sn-W-Ba-O_x_/TiO_2_ and found that TiO_2_ provided sufficient acid sites, CeO_2_ enhanced redox properties, weakened acid strength, and increased chemisorbed oxygen concentration, which helped to promote the activation and desorption of NH_3_. They also reported that SnO_2_ increased chemisorbed oxygen concentration, enhanced redox properties, and improved the activation rate of NH_3_, WO_3_ increased the amount of acid, and BaO enhanced resistance to water vapor and SO_2_ [371]. A rather stable NO conversion was achieved at 350 °C in the presence of 5% H_2_O and 200 ppm SO_2_ [371].

Zeng et al. [346] prepared a P-doped CeO_2_/TiO_2_ catalyst by dispersing CeO_2_ on strongly acidic and highly stable mesoporous P-doped TiO_2_. The P-doped CeO_2_/TiO_2_ catalyst exhibited much higher catalytic activity than the CeO_2_/TiO_2_ catalyst at temperatures of 200–450 °C and a rather stable NO conversion even in the presence of 5% H_2_O and 500 ppm SO_2_ at 200 °C [346]. Mu et al. [354] synthesized an ordered mesoporous CeSnO_x_/TiO_2_ catalyst through a classical evaporation-induced self-assembly (EISA) strategy and ascribed the low-temperature NH_3_-SCR activity to abundant reactive oxygen species (O_α_), improving the surface acidity and redox capacity of the catalyst. This catalyst also showed a rather stable NO conversion even in the presence of 5% H_2_O and 100 ppm SO_2_ at 220 °C [354]. Mu et al. [356] prepared an ordered mesoporous catalyst, CeSnTiO_x_, modified with copper sulfate and revealed that the copper sulfate-modified one increased the reaction sites (redox site and acid site). They reported that Cu species can interfere with the electron cycle of the catalyst and reduce the strong redox performance of the Ce active site, which can effectively inhibit the adsorption of SO_2_, while S-species (from SO_4_^2−^) can change the distribution of acid sites on the catalyst surface and the total acid content to realize a ‘Fast NH_3_-SCR’ reaction [356]. This catalyst also showed a rather stable NO conversion even in the presence of 5% H_2_O and 100 ppm SO_2_ at 240 °C [356]. Liu et al. [377] prepared a series of W-modified Ce-Sn catalysts by the co-precipitation method and reported that the W species regulated the structure by promoting the formation of the Ce-doped tetragonal SnO_2_ (*t*-Sn(Ce)O_2_) active phase while preventing the generation of the Sn-doped cubic CeO_2_ (*c*-Ce(Sn)O_2_) phase. In addition, the highly dispersed W species on the surface of the Ce_1_W_0.24_Sn_2_O_x_ catalyst also coupled with Ce species to form new Ce-O-W active sites [377]. The W modification also promoted the ability of the catalysts to oxidize NO to NO_2_ at 150–300 °C [377]. Liu et al. [378] reported the promotional effect of Ti in Ti-doped Ce– Sn mixed oxide (Ce–Sn–Ti) catalysts on the low-temperature NH_3_-SCR. The appropriate doping Ti formed Sn–O-Ti, Sn–O-Ce and Ti–O-Ce structures, which could increase the content of Ce^3+^ through electron transfer from Sn or Ti to Ce (Ce^3+^ + Ti^4+^ → Ce^4+^ + Ti^3+^ and 2Ce^4+^ + Sn^2+^ → 2Ce^3+^ + Sn^4+^) [378]. The solid solution structure increased specific surface areas, active sites (Ce^3+^), and Lewis acid sites over the Ce_0.6_Sn_2.4_Ti_2_ catalysts [378].

Yang et al. [379] reported that the emergence of Ce^3+^–O–Ce^3+^ structural units induced by Mo doping in Mo-doped CeO_2_ catalysts led to much better NH_3_-SCR performance by achieving low-energy barrier activation of NH_3_ molecules, thereby changing the dominant reaction mechanism in the catalytic reaction. They also observed the same trend in (W)-doped CeO_2_ catalysts, further confirming the pivotal role of Ce^3+^–O–Ce^3+^ structural units [379]. The addition of MoO_3_ improved the activity of the CeO_2_/TiO_2_ catalyst for NH_3_-SCR irrespective of the presence of H_2_O and SO_2_ because the introduction of Mo to the CeO_2_/TiO_2_ catalyst can suppress the adsorption of H_2_O and SO_2_ and the formation of surface sulfate species [365]. The MoO_3_-promoted CeO_2_/TiO_2_ exhibited higher activity than CeO_2_/TiO_2_ even in the co-presence of H_2_O and SO_2_, showing relatively stable NO conversion in the presence of 5% H_2_O and 50 ppm SO_2_ at 300 °C [365]. Liu et al. [380] reported the enhanced NH_3_-SCR performance of MoO_3_/CeO_2_ catalyst by introducing Cu to induce the generation of active Mo=O structure because the adding of Cu into MoO_3_/CeO_2_ catalyst could create unsaturated sites on CeO_2_ for Mo anchor, and the enhanced electron transfer from Mo to Cu would cause the formation of a new terminal Mo=O with highly distorted octahedral geometry, which is a new Lewis acid site for coordinated NH_3_ production. Meanwhile, the added Cu creates the adsorbed site for gaseous NO, and the formed Mo-O-Cu pair center facilitates the transformation of ionic NO_2_^–^ generated from NO adsorption to NO_2_, which is conducive to the ‘Fast NH_3_-SCR reaction’ process [380]. Mo doping to CeO_2_-SiO_2_ mixed-oxide NH_3_-SCR catalyst exhibited high low-temperature NH_3_-SCR activity and superior N_2_ selectivity and resistance to SO_2_/H_2_O poisoning [366]. The Mo-doped CeO_2_-SiO_2_ catalyst showed a rather stable NO conversion even in the presence of 5% H_2_O and 200 ppm SO_2_ at 250 °C [366]. Two elements (Mo and Cu) could be selected for doping into CeO_2_ to promote the NH_3_ adsorption of CeO_2_-based materials by means of DFT calculations [381].

A comparison of NH_3_-SCR activity among CeO_x_ supported on WO_3_ nanorods (WO_3_-NR) and WO_3_ microspheres self-assembled by nanorods (WO_3_-MP), and WO_3_ nanoparticles (WO_3_-NP) revealed that WO_3_-NP enclosed with (0 0 1) facets of the hexagonal WO_3_ showed the best redox ability due to the largest molar ratio of W^5+^/(W^5+^ + W^6+^), the highest concentration of Ce^4+^ on the surface of Ce/WO_3_-NP, the strongest surface redox properties, and the largest molar ratio of O_α_/(O_α_ + O_β_), which was beneficial for NH_3_-SCR activity in the temperature range of 250–400 °C [382]. Hao et al. [103] designed a novel WO_x_/Cu-CeO_2_ oxide catalyst in which the WO_x_ species are highly dispersed on the {111}/{100}-terminated surface of Cu-doped CeO_2_ nanospheres, which exhibits excellent NH_3_-SCR performance over a wide operating temperature window (200–400 °C), as well as good sulfur resistance and N_2_ selectivity. The stable NO conversion was obtained in the presence of 5% H_2_O and 100 ppm SO_2_ at 250 °C [103]. They found that the Cu introduction into the lattice of CeO_2_ not only increased the surface Ce^3+^ and oxygen vacancy concentration but also provided more sites for capture and dispersion of WO_x_ species, thus mediating and improving the Lewis and Brønsted acid sites and reducibility of catalyst [103]. They also found that electronic interaction between WO_x_ and Cu-doped CeO_2_ was enhanced due to the existence of interactions of short-range Ce-O-Cu and Ce-O-W [103]. Fang et al. [383] designed a Ce_1_–W_1_/TiO_2_ model catalyst by anchoring Ce_1_–W_1_ atom pairs on anatase TiO_2_(001) to investigate the synergy between Ce and W in NH_3_-SCR and found that the Ce_1_–W_1_ synergy not only shifted down the lowest unoccupied states of Ce_1_ near the Fermi level, thus enhancing the abilities in adsorbing and oxidizing NH_3_ but also makes the frontier orbital electrons of W_1_ delocalized, thus accelerating the activation of O_2_.

The promotional effect of Nb doping to CeO_2_/TiO_2_ in terms of the resistance to high GHSV, H_2_O, and SO_2_ was ascribed to the better dispersion of Ce, more chemisorbed oxygen species, and Ce^3+^/Ce^4+^ redox pairs on the catalyst surface [361]. The Ce_20_Nb_20_Ti catalyst was slowly deactivated in the presence of 10% H_2_O and 200 ppm SO_2_ at 300 °C [361]. Zhu et al. [360] prepared a series of porous Ce_x_Nb_1-*x*_ (x = 0, 0.2, 0.4, 0.6, 1) hollow nanospheres by a modified seed-mediated growth approach and found that a strong interaction between Nb_2_O_5_ and CeO_2_ affected the oxygen defects, valence, reducibility, and the number of acid sites. The porous Ce_0.4_Nb_0.6_ nanospheres with more acid sites and excellent reducibility exhibited superior catalytic activity in the temperature range of 250–450 °C and high catalytic stability for H_2_O and SO_2_, showing reversible deactivation in the presence of 200 ppm SO_2_ at 300 °C, which can be attributed to the porous double-shell structure and the strong interaction of Nb_2_O_5_ and CeO_2_ species [360]. Jiang et al. [362] synthesized Ce-Nb-Ti metal oxide catalysts (CeNbTi) and found that CeNbTi prepared with the impregnation method possessed superior NH_3_-SCR performance in the temperature range of 250–450 °C and better resistance to SO_2_ and potassium, showing irreversible deactivation in the presence of 500 ppm SO_2_. The best catalyst had relatively high concentrations of Ce^3+^ and chemisorbed oxygen, a strong synergistic effect among different components, and well-dispersed active species [362]. More importantly, the catalyst had stronger reduction capacity and a large number of Lewis acid sites, which contributed to its excellent catalytic performance [362]. Zhang et al. [359] synthesized CeO_2_, Ce–Nb–O_x_ and Nb_2_O_5_ catalysts by the citric acid method and found that the mixed oxide Ce–Nb–O_x_ presented a higher NH_3_-SCR activity than the single oxide CeO_2_ or Nb_2_O_5_ catalyst. In addition, the Ce–Nb–O_x_ catalyst showed high resistance towards H_2_O and SO_2_ at 280 °C [359]. The incorporation of Nb provides abundant oxygen vacancies for capturing more surface adsorbed oxygen, which provides a superior redox capability and accelerates the renewal of active sites. Niobium pentoxide shows high surface acidity, which is partly retained in the Ce–Nb–O_x_ catalyst possessing a high content of Lewis and Brønsted acid sites [359]. Ding et al. [363] synthesized a set of catalysts for the Nb/CeSi_2_ with different loadings of niobium and found that a catalyst for 20Nb/CeSi_2_ exhibited the best low-temperature NH_3_-SCR performance while maintaining excellent SO_2_/H_2_O resistance, showing relatively stable NO conversion in the presence of 5% H_2_O and 200 ppm SO_2_ at 250 °C.

Since the redox properties of ceria can operate at medium and high temperatures, in order to achieve low-temperature NH_3_-SCR activity, catalysts containing ceria should include Cu, Fe, and Mn together in the catalyst composition. Among them, Mn is the most promising metal component for this purpose according to the literature.

### 2.5. Other Catalysts

Generally, the surface concentration of vanadium is an important factor to determine the surface vanadium species such as monomeric vanadyl without V–O–V bonds, oligomeric vanadyl with V–O–V bridge, and crystallized V_2_O_5_ nanoparticles, which can be found at a low surface concentration (<2 V atoms/nm^2^), moderate surface concentration (2–8 V atoms/nm^2^), and high surface density (>8 V atoms/nm^2^), respectively [7]. The polymeric vanadyl species have been reported to have a higher NH_3_-SCR activity than the monomeric vanadyl species because the coupling effect of the polymeric structure shortens the reaction pathway for the regeneration of redox sites [38]. Inomata et al. [384] reported that defective bulk vanadium oxide (V(V) + V(IV)) catalysts, synthesized by the calcination of vanadium(IV)-oxalate at 270 °C, showed NH_3_-SCR activity at a low temperature below 150 °C. The transformation of crystalline V_2_O_5_ on low-vanadium-loading catalysts into an active polymeric vanadyl species under NH_3_-SCR reaction conditions was reported to explain a remarkably enhanced catalytic performance of the vanadia-based catalyst at low temperatures [385]. Highly active supported oligomeric surface vanadia sites, which can provide exclusive centers for adsorbed bridging and bidentate nitrates and assist in NH_3_ activation to generate amide intermediates, could be fabricated by transformation from monomeric surface VO_x_ sites from a classic supported V_2_O_5_–WO_3_/TiO_2_ catalyst via a H_2_ plasma treatment [386]. Lin et al. [387] prepared vanadia supported on defective TiO_2_ nanosheets, which demonstrated excellent NH_3_-SCR performance over a wide temperature range of 140–380 °C. This catalyst was also resistant to H_2_O and SO_2_ poisoning, maintaining an NO conversion at 180 °C [387]. Hwang et al. [388] mechanochemically localized vanadia on the surface of WO_3_-TiO_2_ by physically grinding high-vanadia-loading V_2_O_5_/WO_3_-TiO_2_ with WO_3_-TiO_2_. They found that clustered vanadia sites exhibited enhanced activity for low-temperature (<250 °C) NH_3_-SCR [388]. This catalyst also exhibited superior sulfur resistance at 220 °C because the exposed TiO_2_ sites absorbed ABS from the clustered vanadia sites, preventing the blockage of the catalytic active sites [388].

A comparison of NH_3_-SCR activity among V_2_O_5_, Na_0.33_V_2_O_5_, and Na_1.2_V_3_O_8_ synthesized by the oxalate method revealed that the reaction rate of Na_0.33_V_2_O_5_ was 6.1-times higher than that of V_2_O_5_, indicating that Na intercalation accelerates NH_3_-SCR cycles [389]. Bulk W-substituted vanadium oxide catalysts with the high redox ability and reactivity of Brønsted acid sites were reported to be active for NH_3_-SCR at a low temperature (100–150 °C) and in the presence of water (~20 %). Lewis acid sites of W-substituted vanadium oxide are converted to Brønsted acid sites in the presence of water vapor, and NH_4_^+^ species adsorbed on Brønsted acid sites react with NO with the reduction of V^5+^ sites at 150 °C [390]. Zr-doped CeVO_4_ solid solution catalysts showed excellent NH_3_-SCR performance from 150 to 375 °C because of the increased Brønsted/Lewis acid sites and facilitated electron transfer among Ce, Zr, and V [391]. The Ce_0.85_Zr_0.15_VO_4_ catalyst was slowly deactivated at 190 °C in the presence of 8% H_2_O and 200 ppm SO_2_ [391]. Furthermore, TiO_2_ nanosheets promoted the activity and H_2_O/SO_2_ tolerance compared with TiO_2_ nanoparticles for Zr–CeVO_4_/TiO_2_ catalysts [392]. However, TiO_2_ nanosheets were slowly deactivated at 225 °C in the presence of 8% H_2_O and 200 ppm SO_2_ [392]. FeVO_4_/TiO_2_ was reported to show stable NO conversion in the presence of 200 ppm SO_2_ at 190 °C and reversible deactivation due to 5% H_2_O at 240 °C [393]. Kim et al. [394] prepared various Ce_0.5_RM_0.5_V_1_O_4_ catalysts in which half of Ce in TiO_2_-supported Ce_1_V_1_O_4_ was replaced by RM (Tb, Er, or Yb) and observed the promotive effect anticipated by RM substitution for Ce_0.5_RM_0.5_V_1_O_4_ only at high temperatures. Ce_0.5_Er_0.5_V_1_O_4_ possessed the greatest Lewis acidity/redox feature, thus revealing the best performance at elevated temperatures [394].

Liu et al. [395] prepared a serial of W_a_Co_0.4_TiO_x_ catalysts (a = 0.04, 0.06, 0.08, 0.10) by the sol–gel method, and the W_0.08_Co_0.4_TiO_x_ catalyst achieved the best NH_3_-SCR activity at 190–430 °C and good resistance to H_2_O/SO_2_. However, the W_0.08_Co_0.4_TiO_x_ catalyst was slowly deactivated at 280 °C in the presence of 100 ppm SO_2_ [395]. The doping of W enhanced the acidity and weakened the oxidation capacity of W_a_Co_0.4_TiO_x_, but the high surface acidity, especially the large number of Brønsted acid sites, can compensate for the effect of the reduced oxidation capacity on the low-temperature activity [395].

Phosphates are used in a variety of catalytic reactions due to their excellent thermal stability, proton conductivity, ion exchange, and acidity, and in P-doped CeO_2_/TiO_2_ catalysts, the amorphous CePO_4_ species has been demonstrated to promote NH_3_-SCR activity by activating the NH_4_^+^ species at the Brønsted acid site of the phosphate radical while generating NH_2_ species that react with gaseous NO via the E–R mechanism [12]. The phosphorus is the acid site for NH_3_ adsorption, and CeO_2_ is the redox site. The NH_3_-SCR proceeded according to the L–H mechanism, and the excellent redox properties promoted the ‘Fast NH_3_-SCR’ reaction after the introduction of 3% H_2_O + 100 ppm SO_2_ at 240 °C [396]. The Ce-O-P catalysts prepared with a sol–gel method exhibited enhanced NH_3_-SCR activity due to increased surface acidity and redox capacity and improved SO_2_ tolerance in the presence of 5% H_2_O and 100 ppm SO_2_ at 250 °C due to the effect of SO_2_ capture by abundant reactive oxygen species [397]. Zhao et al. [398] synthesized a series of cerium phosphate catalysts with different crystal phases by hydrothermal method and co-precipitation method. Hexagonal cerium phosphate (CePO_4_-H) showed better NH_3_-SCR activity in the temperature range of 300–500 °C than monoclinic cerium phosphate (CePO_4_-M) and mixed phases of CePO_4_-H and CePO_4_-M because CePO_4_-H had much stronger surface acidity and more surface adsorbed oxygen species [398].

Fe- and Cu-based catalysts have been demonstrated to have enhanced high-temperature NH_3_-SCR activity in the presence of SO_2_ due to the increased acidity derived from the sulfate formed. However, supported sulfate catalysts have the disadvantage of exhibiting low-temperature NH_3_-SCR activity due to their poor redox properties. Therefore, it is necessary to enhance the redox ability of the catalyst to improve its low-temperature NH_3_-SCR activity. The active ingredient, sulfate, can prevent the formation of metal sulfates, but the deposition of AS/ABS with decreasing temperature is the main cause of catalyst deactivation. Therefore, future research on sulfate-based catalysts should focus on improving their redox capacity while promoting the degradation of AS/ABS [399]. NO_x_ reduction conversions and N_2_ selectivity of sulfated CeO_2_ cubes prepared by the impregnation of CeO_2_ cubes by ammonium sulfates could be significantly improved compared with pure CeO_2_ cubes. Sulfation treatment could create and strengthen Brønsted acid sites originated from the protons on surface sulfates, further facilitating ammonia adsorption and activation [400]. Yu et al. [401] prepared CuSO_4_/TiO_2_, Fe_2_(SO_4_)_3_/TiO_2_, MnSO_4_/TiO_2_, Ce(SO_4_)_2_/TiO_2_, and CoSO_4_/TiO_2_ catalysts via a sol–gel protocol. The presence of SO_2_ had little influence on the activity of all metal sulfate catalyst samples [401]. CuSO_4_/TiO_2_ showed the highest NH_3_-SCR activity and apparent activation energy among the metal sulfate catalysts following the sequence of CuSO_4_/TiO_2_ < Fe_2_(SO_4_)_3_/TiO_2_ < MnSO_4_/TiO_2_ < Ce(SO_4_)_2_/TiO_2_ < CoSO_4_/TiO_2_ [401]. The amount of acid sites was the main factor that influenced the catalytic activity of the metal sulfate catalysts [401].

## 3. Strategies to Improve the Low-Temperature NH_3_-SCR Activity

**Figure 7:**
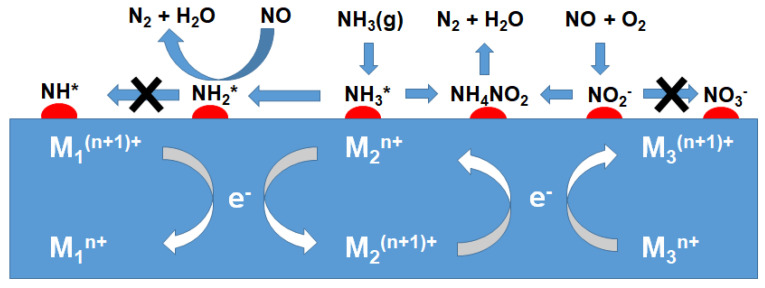
The schematic illustration of the plausible surface reactions during NH_3_-SCR over multicomponent mixed metal oxide catalysts with multiple redox circles to avoid over-oxidation of NH_3_ [12,166].

The reaction mechanism for NH_3_-SCR indicates that both surface acid site and redox property are required to complete the catalytic cycle. Two representative reaction mechanisms have been proposed for this reaction: The L–H and E–R mechanisms, which predominate at low and medium/high temperatures, respectively. In any case, adsorption of NH_3_ on the surface acid sites followed by oxidative dehydrogenation is an important step in this reaction. Therefore, the NH_3_-SCR catalyst surfaces must have specific acid sites that are required for the adsorption of NH_3_ onto the catalyst surface. Lewis and Brønsted acidic sites enable the adsorption of NH_3_ and conversion of NH_3_ to NH_4_^+^, respectively, which is a prerequisite for the generation of the -NH_2_ active species, and its subsequent interaction with NO_2_ and NO species is an essential component of this reaction. The key to broadening the active temperature window of NH_3_-SCR catalysts, including the low-temperature region, is to activate the N-H bond in the adsorbed NH_3_. The oxidative activation of adsorbed NH_3_ is very important. Dehydrogenation of NH_3_ produces the active NH_2_ species, and -NH_2_ can react with gaseous NO or adsorbed NO/NO_2_/nitrate, nitrite, and other species to form N_2_. The oxidation capability of the catalyst surface determines the efficiency of NH_3_ dehydrogenation; therefore, the surface redox sites of the catalyst play an important role. At low temperatures, the adsorption of NH_3_ is not a significant obstacle; rather, the oxidation capacity of the catalyst itself becomes particularly important at low temperatures. However, it is important to note that the surface oxidation of the catalyst should not be too strong, as too much surface oxidation will result in the over-oxidation of NH_3_ to NH or -N, resulting in the consumption of NH_3_ and the production of significant amounts of N_2_O (Figure 7). The over-oxidation of NO_2_ to NO_3_^–^ also adversely affects NH_3_-SCR activity and N_2_ selectivity, since the formation of N_2_O is usually due to the reaction of adsorbed -NH with gas-phase NO or surface-bound NH_4_^+^ with NO_3_^–^ (Figure 7). Therefore, the oxidizing power of the catalyst should be ‘just right,’ meaning that it should have enough oxidizing power to remove the first H from NH_3_, but not over-oxidize NH_3_ to -NH.

Many researchers have observed that low-temperature NH_3_-SCR reactions occur primarily through the L–H mechanism. NO_2_, nitrite, and nitrate species are important intermediates in the L–H mechanism, and the activation of NO to NO_2_ (gas phase or adsorption) is very important. The importance of NO_2_ generation by NO oxidation can also be seen from the fact that NO_2_ contributes to the occurrence of the ‘Fast NH_3_-SCR’ pathway. It is widely recognized that when a certain amount of NO_2_ coexists with NO, the reaction rate increases significantly, resulting in ‘Fast NH_3_-SCR’, which significantly improves NH_3_-SCR performance. Koebel et al. [402,403] first discovered that partial conversion of NO to NO_2_ with the help of an oxidizing catalyst greatly improved the catalytic performance of the NH_3_-SCR, especially at low temperatures. The optimal NO_2_:NO ratio is 1:1, which allows all NO_x_ to react through a ‘Fast NH_3_-SCR’ reaction. The evolution of NO_2_ into nitrate and nitrite after adsorption is also important. Nitrite/nitrate bonding can occur to form monodentate nitrate, linear nitrite, bridging nitrate, and bidentate nitrate. These nitrates/nitrites have specific reactivity at certain temperatures; the level of reactivity depends on the catalyst system. In general, low-stability nitrates/nitrites react with NH_4_^+^ at low temperatures and contribute to enhanced low-temperature activity, whereas high-stability nitrates/nitrites are detrimental to low-temperature activity because they are too stable to react with NH_x_ species and thus poison the active site.

Some promising strategies to improve the acidity and redox properties of metal oxide catalysts have been proposed, such as modification or doping of transition and rare earth metals, optimization of preparation methods, generation of new nanostructures, morphology modification, exposure of specific crystal faces, etc. [12]. As shown in the previous sections, MnO_x_-based catalysts exhibit very good low-temperature NH_3_-SCR activity but always produce a large amount of by-product N_2_O. In order to regulate the redox property and surface acidity of MnO_x_ catalysts, multicomponent Mn-based composite oxides have been reported. They generally contain VO_x_, Fe_2_O_3_, or CuO as the main active metal oxides and Al_2_O_3_, SiO_2_, TiO_2_, CrO_x_, CoO_x_, NiO, Y_2_O_3_, ZrO_2_, NbO_x_, MoO_x_, AgO_x_, SnO_2_, SbO_x_, LaO_x_, CeO_x_, PrO_x_, NdO_x_, SmO_x_, EuO_x_, GdO_x_, DyO_x_, HoO_x_, ErO_x_, TmO_x_, TaO_x_, and WO_3_ as promoters. The creation of a multi redox cycle over a multicomponent oxide catalyst effectively promotes electron transfer and facilitates the adsorption/activation of NO/NH_3_ (Figure 7).

Highly dispersed metal oxide composites can be prepared by utilizing MOF [375,404,405,406] or LDH [216,407,408,409] as precursors and constructing solid solution catalysts. Designing specific nanostructures such as nanoneedles, nanospheres, nanotubes, or core-shell structures can increase the amount of acid sites/reactive oxygen species and promote the formation of active species. Selective exposure of certain facets of the active ingredient, such as MnO_x_ and Fe_2_O_3_, as well as TiO_2_ and CeO_2_ support, can improve redox/acidic properties and metal-support interactions. Additionally, acidification or functionalization of the support with specific acids or oxygen/nitrogen-containing groups can significantly improve acidity and the interaction between the active ingredient and support.

## 4. Strategies to Improve the H_2_O/SO_2_ Tolerance at Low Temperatures

Since water vapor is an unavoidable part of real flue gas, its effect on NH_3_-SCR activity has been an important issue. Positive or negative effects have been reported depending on the reaction conditions (e.g., reaction temperature), catalyst, and reducing agent [410,411]. Although the negative effect of water vapor on NH_3_-SCR activity over low-temperature catalysts has been frequently reported due to its competitive adsorption of NH_3_ and/or NO onto the active sites, regardless of the L–H and E–R mechanisms, irreversible deactivation was seldom reported and reversible deactivation was commonly observed, where initial catalytic activity could be recovered after stopping the introduction of water vapor into the feed [412]. Controlling the surface hydrophobicity of the catalyst by incorporating hydrophobic metal oxide shells into the active site [293] or using hydrophobic supports [33,34] can be effective in minimizing the adverse effects of water at low temperatures.

In contrast to the effect of water vapor, SO_2_ has been reported to have a negative effect on NH_3_-SCR catalytic activity and is known to cause irreversible deactivation, especially for low-temperature catalysts [19,31,412,413,414,415], which limits the practical application of low-temperature catalysts.

There are several strategies to improve the SO_2_ tolerance of catalysts: (1) reducing the adsorption of SO_2_ and its subsequent oxidation; (2) improving the adsorption of NH_3_ and NO to form active intermediates in the presence of surface sulfate species; (3) building sacrificial sites to protect active sites; (4) promoting the decomposition of sulfate; and (5) searching for SO_2_-tolerant compounds with good NH_3_-SCR activity [12].

**Figure 8:**
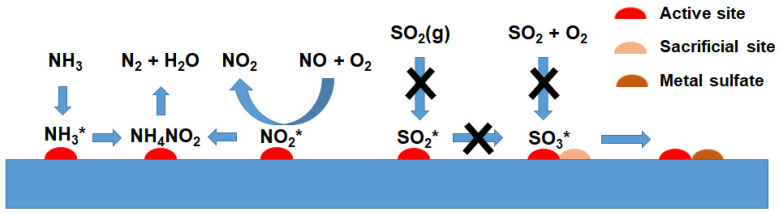
The schematic illustration of the plausible surface reactions during low-temperature NH_3_-SCR in the presence of SO_2_.

(1) Reducing the adsorption of SO_2_ and its subsequent oxidation. Inhibiting the adsorption and oxidation of SO_2_ can significantly reduce the deposition of sulfates. Increasing the acidity of the catalyst can reduce SO_2_ adsorption to some extent. However, on the other hand, more NH_3_ can also be adsorbed on the surface acidic sites, promoting the formation of ammonium sulfate. Therefore, it is important to fine-tune the surface acidity to reduce the adsorption of SO_2_ and NH_3_ simultaneously. The introduction of SiO_2_, Al_2_O_3_, and TiO_2_ to the low-temperature NH_3_-SCR catalysts might be effective for this purpose. Inactive metal sulfates and ammonium sulfates (i.e., AS and ABS) are derived from the reaction of SO_3_ with metal oxides and NH_3_, respectively, so it is necessary to inhibit the oxidation of SO_2_ to SO_3_ by decreasing the oxidation ability of active sites via adding electron-donating promoters [282,310]. Since NO_2_, which can form under ‘Fast NH_3_-SCR’ conditions at low temperatures, can promote the oxidation of SO_2_ to SO_3_, designing a catalyst that can follow the ‘Fast NH_3_-SCR’ while inhibiting the oxidation of SO_2_ to SO_3_ is challenging but necessary to achieve high low-temperature NH_3_-SCR activity as well as SO_2_ tolerance (Figure 8).
(2) Improving the adsorption of NH_3_ and NO to form active intermediates in the presence of surface sulfate species. When the active sites begin to be sulfated by SO_x_, the redox capacity of the active sites is reduced, and this leads to a decrease in NH_3_-SCR activity, especially at low temperatures. Active NO_x_ species are adsorbed competitively with SO_x_, but the adsorption of NH_4_^+^ species is generally enhanced by new Brønsted acid sites derived from the deposited sulfate. Therefore, improving the adsorption of active nitrite/nitrate and gaseous NO_2_ species in the presence of sulfate species is important to achieve SO_2_ durability in NH_3_-SCR reactions that follow the L–H mechanism. Compared with un-doped catalysts, more NH_4_^+^, nitrates, and NO_2_ were formed on Eu-modified Mn/TiO_2_ [278] and Zr-modified Fe–Mn/TiO_2_ [187] catalysts following the L–H mechanism, leading to improved SO_2_ tolerance. If the catalyst follows the E–R mechanism, where the active NH_3_ and NH_4_^+^ species react directly with gaseous NO, this can lead to high SO_2_ tolerance as NO does not need to be competitively adsorbed onto the catalyst surface as nitrate or nitrite. However, the E–R mechanism is not prevalent at low temperatures.
(3) Building sacrificial sites to protect active sites. A rather simple strategy in which H-Y zeolite was physically mixed with V_2_O_5_/TiO_2_ to trap ABS was reported to be effective to protect vanadium active sites, maintaining stable performance in the presence of 10% H_2_O and 30 ppm SO_2_ at a low temperature of 220 °C [416]. Ceria is well known to interact with SO_2_ to form CeSO_4_ or Ce_2_(SO_4_)_3_, which can be used as a sacrificial site to protect the main active phase from sulfation, thereby improving the SO_2_ tolerance of the catalyst [417]. Cr [173] and Nb [188] were also reported to react with SO_2_ preferentially to prevent the sulfuration of active metal oxides (Figure 8). Designing a core–shell structure with outer sacrificial sites for SO_x_ adsorption and ammonium sulfate deposition and inner active sites for NH_3_-SCR can effectively protect active sites from sulfate deposition [17,22,23,216,418,419].
(4) Promoting the decomposition of sulfates. Ammonium sulfate and metal sulfate are two common sulfates observed during NH_3_-SCR in the presence of SO_2_. The former can be deposited on the catalyst and block surface active sites, but can be removed by water washing and heat treatment. However, the thermally stable metal sulfates formed through sulfation of active sites irreversibly deactivate the catalysts. Compared with metal sulfates, ammonium sulfates (AS and ABS) can decompose at lower temperatures. Therefore, reducing the decomposition temperature of ammonium sulfates can improve the SO_2_ tolerance at lower temperatures. It was found that ABS decomposed more easily on SBA-15 with larger pores [330]. Therefore, it can be said that the decomposition of ammonia sulfate can be facilitated via constructing some mesoporous structure. Since metal sulfates are very difficult to decompose at low temperatures, it is desirable to interfere with their formation by adding suitable electrophilic or nucleophilic additives that can interact strongly with SO_4_^2–^ and metal cations [12]. Chen et al. [420] proposed to place a single Mo atom on TiO_2_ to form a Mo-Ti acid-base double site on Mo and Ti that can adsorb NH_4_^+^ and HSO_4_^−^ from ABS, respectively. When NH_4_^+^ is oxidized by surface lattice oxygen on the Mo site, the electrons left on the dual site will transfer to the adsorbed HSO_4_^−^ on the Ti site, releasing SO_2_ at low temperatures. Fe doping [310] and Sb addition [195] were also reported to promote the decomposition of ammonium sulfate and inhibit the formation of MnSO_4_.
(5) Searching for SO_2_-resistant compounds. Some metal sulfates, including CuSO_4_ and FeSO_4_, are known to have improved SO_2_ tolerance, but due to their poor redox properties, they can exhibit NH_3_-SCR activity only at moderate and/or high temperatures. Therefore, it is necessary to improve the redox properties of the catalyst to enhance its low-temperature activity. Nevertheless, the removal of deposited ammonia sulfate is not easy at low temperatures, and the resulting catalyst deactivation is inevitable. Therefore, future research should focus on preventing the deposition of ammonia sulfate at low temperatures or promoting its decomposition even if it is deposited while enhancing its redox ability.

The strategies mentioned above may seem different, but they are closely related. Considering all the above strategies, a promising low-temperature NH_3_-SCR catalyst could have the following properties:

(1) The active site must be composed of a multi-component metal oxide with moderate oxidation capacity not to oxidize SO_2_ to SO_3_, but still retain the oxidation capacity to oxidize NO to NO_2_ and follow the ‘Fast NH_3_-SCR’ pathway. Different catalyst compositions can be screened by DFT calculations that allow for the comparison of competing oxidation reactions, including SO_2_ oxidation and NO oxidation, on the proposed catalyst surface [421,422,423]. The other type of active site is the promoted metal sulfate, which has both a high oxidation capacity to activate adsorbed NO species to follow the L–H reaction mechanism and the ability to decompose ammonium sulfate at low temperatures.
(2) The active site mentioned above should be protected from the adsorption of SO_x_ and/or the ammonium sulfate produced by introducing some kind of sacrificial and/or protective component on or near the surface of the active site. The surface acidity, pore structure, and surface composition of the protective layer must be fine-tuned to take into account the adsorption property of SO_x_ and the decomposition of ammonium sulfate.

## 5. Summary

The redox properties and surface acidity are important factors affecting the catalytic activity of NH_3_-SCR. Ammonia is adsorbed on the Lewis and Brønsted acid sites in the form of NH_3_ and NH_4_^+^, respectively, and is oxidatively dehydrogenated to form active intermediates. NO can react directly with adsorbed intermediates derived from NH_3_ in the E–R mechanism, or in the L–H mechanism prevalent at low temperatures, NO can be adsorbed as is or oxidized to NO_2_ and then adsorbed and react with adsorbed ammonia or ammonia derivatives. Moderate redox properties are very important for low-temperature NH_3_-SCR catalysts, as strong redox properties can reduce N_2_ selectivity due to the formation of unwanted N_2_O, and weak redox properties can reduce the overall denitrification rate.

Water vapor and SO_x_ are often found in flue gas along with NO_x_, and their effect on NH_3_-SCR activity is particularly important at low temperatures. Water vapor in flue gas can cause reversible deactivation because it adsorbs competitively with NO_x_ and ammonia for active sites. SO_2_ in flue gas can adsorb strongly to the active site or oxidize to SO_3_ and further react with ammonia in the presence of water vapor to form ammonium bisulfate and/or ammonium sulfate, which irreversibly blocks the active site. SO_3_ can react directly with active metal oxides to form metal sulfates with weak redox properties, resulting in low NH_3_-SCR activity at low temperatures.

Among Cu-, Fe-, Mn-, and Ce-based catalysts, Mn-containing multivalent metal oxides can be promising candidates for low-temperature NH_3_-SCR. The redox properties of MnO_x_ can be tuned by the incorporation of other multivalent metal oxides. The electronic properties of the active metal can be fine-tuned by adding appropriate promoters. The reduction of NH_3_-SCR activity by water vapor can be overcome by increasing the surface hydrophobicity, which can be accomplished by using a hydrophobic support or by applying a hydrophobic sheath to the outside of the active metal oxide. When selecting the active metal oxide, DFT calculations should be performed to compare SO_2_ adsorption, NO oxidation, and SO_2_ oxidation to ensure that the oxidation of NO occurs without the adsorption of SO_2_ and the oxidation of SO_2_, allowing ‘Fast NH_3_-SCR’ to proceed. The mesoporous shell will not only help to remove ammonium sulfate at the reaction temperature but also prevent SO_2_ poisoning. Metal sulfate catalysts with strong redox properties and V-based catalysts containing oligomeric vanadyl species with sulfur-resistant properties still need to be further investigated for application in NH_3_-SCR at low temperatures.

## Figures and Tables

**Table 1:** NH_3_-SCR activity over some Cu-based catalysts.

Catalysts ^1^	Reaction Conditions						NO_x_ Conversion (%)	Ref.
	NO (ppm)	NH_3_ (ppm)	O_2_ (vol%)	H_2_O (vol%)	Space Velocity	T (°C)		
Cu-LTA	500	500	5	10	100,000 h^−1^	230–500	>90%	[40]
Cu-LTA	500	500	5	10	100,000 h^−1^	165–470	>90%	[41]
Cu-ZSM-5	500	575	4	5	30,000 h^−1^	175–375	>90%	[42]
Cu-ZSM-5	1000	1000	3	-	50,000 h^−1^	200–375	~100%	[43]
Cu-ZSM-5	500	500	5	10	100,000 h^−1^	200–400	>90%	[44]
Cu-Beta	1000	1000	6	-	300,000 h^−1^	250–325	>90%	[45]
Cu/BEA	400	400	8	5	30,300 h^−1^	225–475	>90%	[46]
Cu-SSZ-13	500	500	10	5	80,000 h^−1^	160–500	>90%	[47]
Cu-SSZ-13	500	500	5	5	400,000 h^−1^	210–520	>90%	[48]
Cu-SSZ-16	500	500	10	-	42,500 mL·h^−1^·g^−1^	190–440	>90%	[49]
Cu-SSZ-39	500	500	5	5	250,000 h^−1^	225–500	>90%	[50]
Cu-SSZ-52	500	500	5	5	240,000 h^−1^	200–550	>90%	[51]
Cu-SAPO-34	500	500	6.1	6.4	300,000 h^−1^	190~500	>90%	[52]
Cu-RTH	500	500	5	10	100,000 h^−1^	470–750	>90%	[53]
Cu-ERI	300	300	5	3	50,000 h^−1^	240–500	>90%	[54]
Cu-UZM-9	500	500	5	10	100,000 h^−1^	250–650	>90%	[55]
Cu-UZM-35	500	500	5	10	100,000 h^−1^	200–420	>90%	[56]
Cu-ZJM-7	500	500	5	5	80,000 h^−1^	190~550	>90%	[57]
Cu-SAPO-18	1000	1100	5	10	30,000 h^−1^	250–500	>90%	[58]
Cu-SAPO-18	500	500	14	5	130,000 h^−1^	210–540	>80%	[59]
Cu/SSZ-13@SiC	500	500	10	5	80,000 h^−1^	200–360	>90%	[60]
CuFe/BEA	200	200	10	5	40,000 h^−1^	225–375	>90%	[61]
Fe/Cu-SSZ-13	500	500	5	-	50,000 h^−1^	150–500	>90%	[62]
CuY-SAPO-34	350	350	8	5	30,000 h^−1^	200–310	>90%	[63]
CuNd/SAPO-34	200	200	10	5	40,000 h^−1^	200–400	>90%	[64]
Cu-Ce-La-SSZ-13	500	500	5	-	150,000 h^−1^	175–400	>90%	[65]
Ce-Cu-SAPO-18	500	500	14	5	130,000 h^−1^	250–500	>90%	[66]
La-Cu-SAPO-18	500	500	5	-	20,000 mL·h^−1^·g^−1^	250~450	>90%	[67]
La-Cu-SAPO-18	500	500	14	5	130,000 h^−1^	210–580	>80%	[59]
Ce-Cu-SAPO-18	500	500	14	5	130,000 h^−1^	200–600	>90%	[59]
Nd-Cu-SAPO-18	500	500	14	5	130,000 h^−1^	250–550	>90%	[59]
Gd-Cu-SAPO-18	500	500	14	5	130,000 h^−1^	300–460	>90%	[59]
Tb-Cu-SAPO-18	500	500	14	5	130,000 h^−1^	220–570	>90%	[59]
Ho-Cu-SAPO-18	500	500	14	5	130,000 h^−1^	250–550	>90%	[59]
Lu-Cu-SAPO-18	500	500	14	5	130,000 h^−1^	250–520	>90%	[59]
Cu-Ce-USY	500	500	5	-	48,000 h^−1^	180~250	>90%	[68]
CuO(111)/TiO_2_	500	500	5	-	45,000 h^−1^	235–285	>90%	[69]
Cu/ZrO_2_	600	600	10	5	58,333 h^−1^	200–360	>90%	[70]
LaCuO_3-x_/meso-Al_2_O_3_	500	500	3	10	100,000 h^−1^	220–275	>50%	[71]
CuAl-(LDO)/(CNTs)	600	600	5	-	45,000 h^−1^	176–275	>90%	[72]
meso-Cu-SSZ-13@meso-Al-Si	500	500	5	5	400,000 h^−1^	250–520	>90%	[73]
Cu-SAPO-34@Fe-MOR	500	500	5	-	50,000 h^−1^	375–525	>90%	[74]
Cu-SSZ13@Ce_0.75_Zr_0.25_O_2_	500	500	5	-	60,000 h^−1^	220–480	>90%	[75]
Cu-SSZ-13 HN	500	500	3	-	120,000 h^−1^	200~550	>90%	[76]

^1^ LDO: layered double oxide; USY: ultrastable Y; CNTs: carbon nanotubes; meso: mesoporous; Al-Si: aluminosilicate; HN: hollow nanocube.

**Table 2:** NH_3_-SCR activity in the presence of H_2_O and/or SO_2_ over some Cu-based catalysts.

Catalysts ^1^	Reaction Conditions						NO_x_ Conversion (%)	Effects of H_2_O/SO_2_	Ref
	NO (ppm)	NH_3_ (ppm)	O_2_ (vol%)	H_2_O (vol%)	Space Velocity	T (°C)			
Cu-SAPO-18	500	500	14	5	130,000 h^−1^	200–550	>80%	Reversible inhibition with 5% H_2_O and 100 ppm SO_2_ at 300 °C	[97]
Cu-Ce-SAPO-18	500	500	14	5	130,000 h^−1^	200–600	>90%	Reversible inhibition with 5% H_2_O and 100 ppm SO_2_ at 300 °C	[97]
Cu/TNU-9	500	500	5	10	10,000 h^−1^	237–400	>90%	Stable NO_x_ conversion with 10% H_2_O and 100 ppm SO_2_ at 250 °C	[98]
Cu–Ce/TNU-9	500	500	5	10	10,000 h^−1^	225–400	>90%	Stable NO_x_ conversion with 10% H_2_O and 100 ppm SO_2_ at 250 °C	[98]
Cu–Ce–La/TNU-9	500	500	5	10	10,000 h^−1^	200–425	>90%	Stable NO_x_ conversion with 10% H_2_O and 100 ppm SO_2_ at 250 °C	[98]
Mn-Ce/Cu-SSZ-13	500	500	3	3	50,000 h^−1^	125–450	>90%	Reversible inhibition with 3% H_2_O and 100 ppm SO_2_ at 300 °C	[99]
CuSbTiO_x_	700	700	4	4	60,000 h^−1^	250–300	>85%	Slowly deactivated with 5% H_2_O and 150 ppm SO_2_ at 250 °C	[100]
CuO@Cu_3_(BTC)_2_	600	600	4	-	60,000 h^−1^	180–240	>80%	Stable NO_x_ conversion with 4% H_2_O and 150 ppm SO_2_ at 200 °C	[101]
Cu_0.5_Ce_0.5_W_5_O_x_	500	500	5	-	36,000 h^−1^	270–390	~100%	Stable NO_x_ conversion with 5% H_2_O and 50 ppm SO_2_ at 240 °C	[102]
WO_x_/Cu-CeO_2_	500	500	5	-	60,000 h^−1^	220–400	>90%	Stable NO_x_ conversion with 10% H_2_O and 100 ppm SO_2_ at 250 °C	[103]
Nb_0.05_CuCeTi	600	600	3	5	40,000 h^−1^	160–360	>90%	Reversible inhibition with 5% H_2_O and 50 ppm SO_2_ at 250 °C	[104]
CuCeNbO_x_	600	600	5	-	108,000 h^−1^	185–360	>90%	Stable NO_x_ conversion with 5% H_2_O and 100 ppm SO_2_ at 250 °C	[105]
Cu–HPMo/TiO_2_	500	500	8	-	15,000 h^−1^	150–350	>80%	100% NO_x_ conversion with 4% H_2_O and 200 ppm SO_2_ at 200 °C	[106]
Cu–Ce–La/SSZ-13	500	500	5	3	150,000 h^−1^	210–450	>90%	Stable NO_x_ conversion with 10% H_2_O and 100 ppm SO_2_ at 300 °C	[107]
Cu/(ZSM-5@CeO_2_)	1000	1000	8	5	50,000 h^−1^	225–550	>95%	Stable NO_x_ conversion with 5% H_2_O and 200 ppm SO_2_ at 275 °C	[108]
Cu–Ce–La/SSZ-13@ZSM-5	500	500	5	3	150,000 h^−1^	200–450	>80%	Stable NO_x_ conversion with 10% H_2_O and 100 ppm SO_2_ at 300 °C	[107]
Cu-SSZ-13@Ce-MnO_x_/MS	500	500	5	3	150,000 h^−1^	175–475	>90%	Relatively stable NO_x_ conversion with 10% H_2_O and 100 ppm SO_2_ at 250 °C	[109]

^1^ BTC: 1,3,5-Benzenetricarboxylic acid; MS: mesoporous silica.

**Table 3:** NH_3_-SCR activity in the presence of H_2_O and/or SO_2_ over some Fe-based catalysts.

Catalysts	Reaction Conditions						NO_x_ Conversion (%)	Effects of H_2_O/SO_2_	Ref.
	NO (ppm)	NH_3_ (ppm)	O_2_ (vol%)	H_2_O (vol%)	Space Velocity	T (°C)			
Defective α-Fe_2_O_3_	500	500	5.3	-	50,000 h^−1^	250–300	~100%	100% NO_x_ conversion with 5% H_2_O and 50 ppm SO_2_ at 300 °C	[120]
MnFeO_x_	500	500	5	5	75,000 h^−1^	75–275	~100%	A rather stable NO_x_ conversion with 5% H_2_O and 50 ppm SO_2_ at 100 °C	[121]
Ce/α-Fe_2_O_3_	500	500	5	5	90,000 h^−1^	175–325	>95%	Stable NO_x_ conversion wit 5% H_2_O and 200 ppm SO_2_ at 250 °C	[124]
Fe–Ce/TiO_2_	1000	1000	3	-	30,000 h^−1^	175–350	>90%	Slowly deactivated with 500 ppm SO_2_ at 250 °C	[129]
Mo_0.4_Ce_0.3_FeO_x_	2000	2000	8	-	32,000 h^−1^	200–350	>95%	Stable NO_x_ conversion with 10% H_2_O and 200 ppm SO_2_ at 250 °C	[130]
Fe–Ce–W oxides	450	450	2.5	5	20,000 h^−1^	250–500	>90%	Relatively stable NO_x_ conversion with 5% H_2_O and 200 ppm SO_2_ at 350 °C	[131]
Sm/Fe_2_O_3_	500	500	5	5	14,400 h^−1^	175–350	~100%	Stable NO_x_ conversion with 5% H_2_O and 100 ppm SO_2_ at 275 °C	[125]
WO_x_/Fe_2_O_3_	500	500	5	-	50,000 h^−1^	300–425	>90%	Stable NO_x_ conversion with 100 ppm SO_2_ at 300 °C	[128]
H_3_PW_12_O_40_-Fe_2_O_3_	1000	1100	6	-	13,200 h^−1^	300–500	>90%	Stable NO_x_ conversion with 10% H_2_O and 200 ppm SO_2_ at 280 °C	[132]
Mn-W-Sb modified siderite	500	500	5	-	30,000 h^−1^	175–375	>90%	Irreversible deactivation with 5% H_2_O and 100 ppm SO_2_ at 210 °C	[133]
Fe_2_O_3_-promoted halloysite-supported CeO_2_-WO_3_	500	500	5	-	40,000 h^−1^	275–420	>95%	Relatively stable NO_x_ conversion with 8% H_2_O and 100 ppm SO_2_ at 300 °C	[134]
Fe_2_O_3_-CeO_2_@Al_2_O_3_	500	500	5	-	20,000 mL·g^−1^·h^−1^	250–430	>90%	Stable NO_x_ conversion with 10% H_2_O and 500 ppm SO_2_ at 270 °C	[135]
Fe-ZSM-5@CeO_2_	500	500	5	-	177,000 h^−1^	250–425	>90%	Stable NO_x_ conversion with 10% H_2_O and 100 ppm SO_2_ at 350 °C	[136]
Fe-Beta@CeO_2_	500	500	3	5	50,000 h^−1^	225–575	>90%	Stable NO_x_ conversion with 5% H_2_O and 100 ppm SO_2_ at 300 °C	[137]

**Table 4:** NH_3_-SCR activity in the presence of H_2_O and/or SO_2_ over some unsupported Mn-based catalysts.

Catalysts ^1^	Reaction Conditions						NO_x_ Conversion (%)	Effects of H_2_O/SO_2_	Ref.
	NO (ppm)	NH_3_ (ppm)	O_2_ (vol%)	H_2_O (vol%)	Space Velocity	T (°C)			
MnO_x_	500	500	5	-	140,000 h^−1^	125–200	>90%	Relatively stable NO_x_ conversion with 50 ppm SO_2_ at 175 °C	[161]
MnO_x_-SiO_2_	1000	1000	10	-	24,000 h^−1^	150–225	>90%	Relatively stable NO_x_ conversion with 100 ppm SO_2_ at 225 °C	[221]
Mn-TiO_x_	500	500	5	-	100,000 h^−1^	160–370	>90%	Relatively stable NO_x_ conversion with 5% H_2_O at 140 °C. Relatively stable NO_x_ conversion with 100 ppm SO_2_ at 260 °C.	[222]
Cr-MnO_x_	1000	1000	3	-	30,000 h^−1^	115–220	>90%	Reversible inhibition with 100 ppm SO_2_ at 120 °C	[223]
CrMn_2_O_4_ spinel	500	500	5	-	32,000 h^−1^	80–225	>90%	Relatively stable NO conversion with 10% H_2_O and 150 ppm SO_2_ at 200 °C	[173]
MnO_x_-Fe	1000	1000	3	5	30,000 h^−1^	93–220	>90%	Relatively stable NO conversion with 5% H_2_O and 100 ppm SO_2_ at 200 °C	[224]
MnFeCo-LDO	550	550	5	-	30,600 h^−1^	63–400	>90%	Stable NO conversion with 5% H_2_O and 100 ppm SO_2_ at 120 °C	[225]
Mn-Fe-Mg oxides	1000	1000	4	-	30,000 h^−1^	125–200	>90%	Stable NO_x_ conversions with 3% H_2_O and 100 ppm SO_2_ at 150 °C	[226]
Mn-Fe-Al oxides	500	500	5	-	60,000 h^−1^	110–250	>90%	Stable NO_x_ conversions with 100 ppm SO_2_ at 150 °C	[227]
Mn-Fe-Ce oxides	500	500	11	-	36,000 h^−1^	180–277	>90%	Stable NO_x_ conversions with 100 ppm SO_2_ at 225 °C	[228]
Mesoporous Mn-Fe-Ce-Ti oxides	600	480	2	-	24,000 h^−1^	210–395	>90%	Reversible inhibition with 300 ppm SO_2_ at 240 °C	[229]
Mn_2_Co_1_O_x_	1000	1000	5	-	30,000 h^−1^	150–325	>90%	Reversible inhibition with 10% H_2_O and 100 ppm SO_2_ at 200 °C	[230]
Mn-Co oxides	500	500	5	-	23,000 h^−1^	100–300	>90%	Reversible inhibition with 8% H_2_O at 120 °C	[231]
Mn-Co oxides	500	500	3	-	30,000 h^−1^	100–280	>90%	Stable NO_x_ conversions with 5% H_2_O and 100 ppm SO_2_ at 160 °C	[232]
Mn-Co oxides	500	500	5	-	140,000 h^−1^	80–200	>90%	Stable NO_x_ conversions with 5% H_2_O and 50 ppm SO_2_ at 125 °C	[233]
Mn-Co oxides	500	500	5	-	50,000 h^−1^	60–300	>90%	Reversible inhibition with 5% H_2_O and 100 ppm SO_2_ at 200 °C	[234]
Co_1_Mn_4_Ce_5_O_x_	500	500	5	-	48,000 h^−1^	80–175	>90%	Relatively stable NO_x_ conversion with 10% H_2_O and 150 ppm SO_2_ and at 175 °C	[235]
MnCoVO_x_	500	500	5	-	60,000 mL·g^−1^·h^−1^	175–425	>90%	Relatively stable NO conversion with 5% H_2_O and 100 ppm SO_2_ at 200 °C	[236]
Mn-Ni oxides	550	550	5	-	64,000 h^−1^	105–275	>90%	Stable NO_x_ conversion with 100 ppm SO_2_ at 230 °C	[237]
NiMn_2_O_4_	500	500	5	-	32,000 h^−1^	73–250	>98%	Stable NO_x_ conversions in the presence of 150 ppm SO_2_ at 175 °C	[238]
Ni_1_Mn_4_O_5_	500	500	5	-	48,000 h^−1^	125–200	>90%	Relatively stable NO_x_ conversion with 10% H_2_O and 150 ppm SO_2_ at 175 °C	[235]
Ni_1_Mn_2_O_4_-S	500	500	5	-	68,000 h^−1^	90–230	>90%	Relatively stable NO_x_ conversion with 5% H_2_O and 100 ppm SO_2_ at 150 °C	[239]
Ni_1_Mn_0.5_Al_0.5_O_x_	500	500	5	5	60,000 h^−1^	100–250	>90%	Relatively stable NO conversion with 5% H_2_O and 100 ppm SO_2_ at 200 °C	[240]
Ni_a-x_Mn_x_AlO_y_	500	500	6.5	5	45,000 h^−1^	120–225	>90%	Deactivation with 5% H_2_O and 100 ppm SO_2_ at 210 °C	[241]
Mn-Ni-Ti oxides	500	500	5	-	40,000 h^−1^	320–460	>90%	Relatively stable NO_x_ conversion with 10% H_2_O and 100 ppm SO_2_ at 400 °C	[242]
Mn-Ni-Ti oxides	1000	1000	3	-	40,000 h^−1^	190–360	~100%	Relatively stable NO_x_ conversion with 15% H_2_O and 100 ppm SO_2_ at 240 °C	[243]
Ni_0.5_Mn_0.5_Fe_0.5_O_x_	500	500	5	-	60,000 h^−1^	100–300	>80%	Relatively stable NO_x_ conversion with 5% H_2_O and 100 ppm SO_2_ at 200 °C	[244]
Mn-Zr oxides	1000	1000	3	-	30,000 h^−1^	100–200	~100%	Reversible deactivation with 5% H_2_O and 100ppm SO_2_ at 150 °C	[245]
Mn-Zr-Ti oxides	650	650	5	-	36,000 h^−1^	160–300	>90%	Relatively stable NO_x_ conversion with 3% H_2_O and 50 ppm SO_2_ at 180 °C	[246]
Mn_2_Nb_1_O_x_	500	500	5	-	50,000 h^−1^	120–200	>90%	Irreversible deactivation with 100 ppm SO_2_ at 200 °C	[247]
Mn-Ce oxides	1000	1000	2	-	35,000 h^−1^	100–300	>80%	Relatively stable NO_x_ conversion with 12% H_2_O and 100 ppm SO_2_ at 110 °C	[248]
Mn-Ce oxides	3000	3000	15	-	60,000 mL·g^−1^·h^−1^	100–250	>80%	Deactivation with 5% H_2_O and 100 ppm SO_2_ at 175 °C	[206]
Mn-Ce nanowire aerogel	500	500	5	-	32,000 h^−1^	100–400	>90%	Stable NO_x_ conversion with 10% H_2_O and 250 ppm SO_2_ at 150 °C	[249]
Ce-Ti/MnO_2_	600	600	6	-	40,000 h^−1^	100–225	>95%	Relatively stable NO_x_ conversion with 10 vol% H_2_O and 50 ppm SO_2_ at 150 °C	[250]
Mn–Ce–Ti oxides	500	500	5	-	14,400 h^−1^	170–320	>90%	Stable NO conversion with 5 vol% H_2_O and 100 ppm SO_2_ at 200 °C	[251]
Mn-Ce-Ti oxides	500	500	5	-	64,000 h^−1^	150–350	>90%	Stable NO conversion with 5 vol% H_2_O and 50 ppm SO_2_ at 200 °C	[252]
Mn-Sn-Ce oxides	1000	1000	2	-	35,000 h^−1^	75–250	>90%	Stable NO conversion with 9% H_2_O and 100 ppm SO_2_ at 220 °C	[194]
Mn-Sn-Ce oxides	1000	1000	2	-	35,000 h^−1^	75–225	~100%	Relatively stable NO_x_ conversion with 12% H_2_O and 100 ppm SO_2_ at 110 °C	[248]
Mn-Sn−Ce oxides	500	500	5	-	60,000 mL·g^−1^·h^−1^	175–275	>60%	Stable NO conversion with 5% H_2_O and 100 ppm SO_2_ at 250 °C	[253]
Mn-Pr-Ce oxides	600	600	5	-	108,000 h^−1^	150–400	>80%	Stable NO conversion with 5% H_2_O and 100 ppm SO_2_ at 250 °C	[205]
Mn-Sm oxides	500	500	5	-	48,600 h^−1^	50–250	>90%	Stable NO conversion with 2% H_2_O and 100 ppm SO_2_ at 250 °C	[254]
Mn-Sm oxides	500	500	5	-	60,000 mL·g^−1^·h^−1^	200–325	>80%	Deactivation with 5% H_2_O and 100 ppm SO_2_ at 175 °C	[206]
Mn-Sm-Ti oxides	500	500	5	-	36,000 h^−1^	150–300	>90%	Stable NO conversion with 5% H_2_O and 100 ppm SO_2_ at 200 °C	[255]
Mn-Sm-Ti oxides	500	500	5	-	50,000 h^−1^	75–230	>90%	Stable NOx conversion with 5% H_2_O and 100 ppm SO_2_ at 100 °C	[256]
Mn-Sm-Fe oxides	500	500	5	-	60,000 h^−1^	75–200	~100%	Relatively stable NO_x_ conversion with 5% H_2_O and 100 ppm SO_2_ at 200 °C	[257]
Mn-Sm-Zr-Ti oxides	500	500	5	-	30,000 h^−1^	125–275	~100%	Relatively stable NO_x_ conversion with 2.5% H_2_O and 100 ppm SO_2_ at 200 °C	[258]
Mn-Sm-Ce-Ti oxides	500	500	5	-	80,000 h^−1^	150–400	>90%	Relatively stable NO conversion with 5% H_2_O and 200 ppm SO_2_ at 200 °C	[259]
Mn-Nd oxides	500	500	5	-	60,000 mL·g^−1^·h^−1^	125–230	>90%	Relatively stable NO conversion 5% H_2_O and 100 ppm SO_2_ at 175 °C	[206]
Mn-Eu oxides	600	600	5	-	108,000 h^−1^	130–400	~100%	Relatively stable NO conversion with 5% H_2_O and 100 ppm SO_2_ at 350 °C	[260]
Mn-Eu-Ce oxides	500	500	10	10	60,000 h^−1^	100–250	>90%	Stable NO_x_ conversions with 10% H_2_O and 50 ppm SO_2_ at 230 °C	[210]
Mn-Gd oxides	500	500	5	-	100,000 h^−1^	120–330	~100%	Stable NO_x_ conversions with 100 ppm SO_2_ at 200 °C	[261]
Mn-W-Ce oxides (W_0.1_Mn_0.4_Ce_0.5_)	500	500	5	-	300,000 h^−1^	150–270	>90%	Stable NO_x_ conversions with 60 ppm SO_2_ at 175 °C	[262]
BiMnO_3_	1000	1000	5	-	10,000 h^−1^	160–250	>80%	Relatively stable NO_x_ conversions with 5% H_2_O and 100 ppm SO_2_ at 140 °C	[220]

^1^ LDO: layered double oxide.

**Table 5:** NH_3_-SCR activity in the presence of H_2_O and/or SO_2_ over some supported Mn-based catalysts.

Catalysts ^1^	Reaction Conditions					NO_x_ Conversion (%)	Effects of H_2_O and SO_2_	Ref.
	NO (ppm)	NH_3_ (ppm)	O_2_ (vol%)	Space Velocity	T (°C)			
Mn/Fe-Ti spinel	500	500	2	24,000 mL·g^−1^·h^−1^	150–250	>95%	Stable NO_x_ conversion with 8% H_2_O and 60 ppm SO_2_ at 200 °C	[263]
Mn/ZrO_2_-TiO_2_	500	500	4	35,000 h^−1^	175–350	>97%	Irreversible deactivation with 10% H_2_O and 200 ppm SO_2_ at 200 °C	[264]
Mn/CeO_2_-TiO_2_	200	220	8	60,000 h^−1^	180–250	>90%	Deactivation with 6% H_2_O and 100 ppm SO_2_ at 180 °C	[265]
Mn/CeO_2_-ZrO_2_	600	660	6	45,000 h^−1^	120–220	>90%	Stable NO_x_ conversion with 3% H_2_O and 100 ppm SO_2_ at 180 °C	[266]
Mn/CeO_2_-ZrO_2_-Al_2_O_3_	1000	1000	5	10,000 h^−1^	150–300	>90%	Relatively stable NO_x_ conversion with 10% H_2_O and 100 ppm SO_2_ at 200 °C	[267]
MnO_x_(0.25)/CoSn_3_Al-LDO	500	500	5	30,000 h^−1^	100–350	>95%	Irreversible deactivation with 5% H_2_O and 150 ppm SO_2_ at 200 °C	[268]
MnCrO_x_/Sepiolite	1000	1000	5	45,000 h^−1^	125–340	>90%	Relatively stable NO_x_ conversion with 200 ppm SO_2_ at 220 °C	[269]
TiO_2_-MnO_x_/CeO_2_-ZrO_2_	500	500	5	60,000 mL·g^−1^·h^−1^	175–225	>90%	Relatively stable NO_x_ conversion with 5% H_2_O and 100 ppm SO_2_ at 200 °C	[171]
Fe-Mn/TiO_2_	1000	1000	10	30,000 h^−1^	100–330	>90%	Stable NO_x_ conversion with 100 ppm SO_2_ at 125 °C	[270]
Fe-Mn/Ti-Zr	1000	1000	4	30,000 h^−1^	80–180	>90%	Deactivation with 6% H_2_O and 100 ppm SO_2_ at 150 °C	[187]
Fe-Mn-Ce/TiO_2_	600	600	3	50,000 h^−1^	155–260	>90%	Stable NO_x_ conversion with 3% H_2_O at 140/180 °C	[271]
Ni-Mn/TiO_2_	400	400	2	50,000 h^−1^	187–240	>90%	Superior H_2_O durability at 200 °C with 10% H_2_O	[272]
Zr-Mn/attapulgite	600	600	3	40,000 h^−1^	153–250	>90%	Stable NO_x_ conversion with 300 ppm SO_2_ at 200 °C	[273]
xSb-4Ce-10Mn/TiO_2_	500	500	3	75,000 mL·g^−1^·h^−1^	135–325	>95%	Deactivation with 5% H_2_O and 100 ppm SO_2_ at 200 °C	[195]
Ce-Mn-TNTs	500	500	5	30,000 h^−1^	150–425	>90%	Stable NO_x_ conversion with 10% H_2_O and 100 ppm SO_2_ at 280 °C	[274]
Ce-Mn/TiO_2_	800	800	3	40,000 h^−1^			Deactivation with 3% H_2_O and 100 ppm SO_2_ at 150 °C	[275]
Ce-Mn/TiO_2_	720	800	3	30,000 h^−1^	120–200	>90%	Deactivation with 100 ppm SO_2_ at 180 °C	[276]
Ce-Mn-V-W/TiO_2_	1500	1500	3	40,000 h^−1^	150–400	>90%	Stable NO_x_ conversion with 5% H_2_O and 100 ppm SO_2_ at 250 °C	[277]
Nd-Mn/TiO_2_	600	600	3	40,000 h^−1^	100–320	100%	Stable NO_x_ conversion with 3% H_2_O and 50 ppm SO_2_ at 120 °C	[278]
Eu-Mn/TiO_2_	600	600	5	108,000 h^−1^	175–400	>90%	Deactivation with 100 ppm SO_2_ at 150 °C	[279]
Ho-Mn-Ce/TiO_2_	800	800	5	10,000 h^−1^	140–220	>90%	Deactivation with 10% H_2_O and 300 ppm SO_2_ at 180 °C	[280]
Ho-Fe-Mn/TiO_2_	800	800	5	20,000 h^−1^			Stable NO_x_ conversion with 15% H_2_O and 200 ppm SO_2_ at 120 °C	[215]
Gd-MnO_x_/ZSM-5	800	800	5	40,000 h^−1^	110–240	>90%	Relatively stable NO conversion with 100 ppm SO_2_ at 180 °C	[212]
Ce_0.5_/Co_1_Mn_0.5_Al_0.5_O_x_	500	500	5		100–250	>90%	Deactivation with 100 ppm SO_2_ at 200 °C	[281]
Mn-Ce-V/AC	500	500	5	18,000 h^−1^	125–300	>95%	Deactivation with 10% H_2_O and 100 ppm SO_2_ at 200 °C	[282]
Nb_2_O_5_-Zn-Ce-Mn/AC	500	500	11	14,500 h^−1^	150–250	>90%	Deactivation with 100 ppm SO_2_ at 200 °C	[189]
NiMnO_x_/activated coke	500	500	10	30,000 h^−1^	125–250	>80%	Stable NO_x_ conversion with 200 ppm SO_2_ at 200 °C	[283]
La-Mn-Fe/activated coke	500	500	5	6000 h^−1^	150–300	>90%	Reversible deactivation with 5% H_2_O and 200 ppm SO_2_ at 150 °C	[284]
Mn/BC	600	600	11	12,000 h^−1^	160–240	>80%	Reversible deactivation with 100 ppm SO_2_ at 180 °C	[285]
Zr-Mn/BC	500	500	5	36,000 h^−1^	125–250	>75%	Stable NO_x_ conversion with 5% H_2_O and 100 ppm SO_2_ at 200 °C	[286]
MnO_x_@CNTs	500	500	3	30,000 h^−1^	165–325	>90%	100% NO_x_ conversion with 4% H_2_O at 225 °C	[287]
MnO_x_/Functionalized multi-walled CNTs	900	900	5	30,000 h^−1^	150–300	>80%	Deactivation with 2.5% H_2_O and 100 ppm SO_2_ at 200 °C	[288]
MnO_x_-CeO_x_/CNTs	400	400	3	12,000 h^−1^	200–260	>90%	Stable NO_x_ conversion with 100 ppm SO_2_ at 220 °C	[289]
MnO_x_-CeO_x_@CNTs	500	500	3	10,000 h^−1^	200–350	>90%	Stable NO_x_ conversion with 4% H_2_O and 100 ppm SO_2_ at 300 °C	[290]
MnCe/granular AC-CNTs	500	550	5	10,000 h^−1^	125–200	>80%	Stable NO_x_ conversion with 5% H_2_O and 50 ppm SO_2_ at 150 °C	[291]
MnO_x_-CeO_2_(8:1)/graphene	500	500	5	24,000 h^−1^	80–140	>99%	Reversible deactivation with 10% H_2_O and 200 ppm SO_2_ at 140 °C	[292]

^1^ LDO: layered double oxide; TNTs: titanium nanotubes; AC: active carbon; BC: biochar; CNTs: carbon nanotubes.

**Table 6:** NH_3_-SCR activity in the presence of H_2_O and/or SO_2_ over some Mn-based core-shell catalysts.

Catalysts ^1^	Reaction Conditions					NO_x_ Conversion (%)	Effects of H_2_O/SO_2_/	Ref.
	NO (ppm)	NH_3_ (ppm)	O_2_ (vol%)	Space Velocity	T (°C)			
MnO_x_@TiO_2_	500	500	5	24,000 h^−1^	110–260	>90%	Deactivation with 10% H_2_O and 200 ppm SO_2_ at 160 °C	[293]
MnO_x_@TiO_2_	500	500	5	30,000 h^−1^	130–375	>90%	Reversible inhibition with 5% H_2_O at 180 °C	[294]
MnO_x_–CeO_2_@TiO_2_	500	500	5	24,000 h^−1^	150–210	>90%	Reversible inhibition with 200 ppm SO_2_ at 180 °C	[295]
MnO_x_@PrO_x_	800	800	5	40,000 h^−1^	93–240	>90%	Irreversible inhibition with 10% H_2_O and 100 ppm SO_2_ at 160 °C	[296]
MnO_x_@CeO_2_	800	800	5	40,000 h^−1^	114–220	>90%	Deactivation with 100 ppm SO_2_ at 220 °C	[297]
α-MnO_2_@CeO_2_	500	500	3	100,000 mL·g^−1^·h^−1^	125–250	>90%	Deactivation with 200 ppm SO_2_ at 220 °C	[298]
MnFe@CeO_x_	500	500	5	30,000 h^−1^	160–240	>90%	Reversible inhibition with 5% H_2_O at 160 °C	[299]
MnO_x_@Eu-CeO_x_	600	600	2.5	90,000 h^−1^	100–210	>90%	Reversible inhibition with 100 ppm SO_2_ at 200 °C	[300]
MnO_x_@Fe_2_O_3_/TNT	500	500	5	40,000 h^−1^	180–360	~100%	Deactivation with 150 ppm SO_2_ at 240 °C	[301]
Ho-TNT@Mn	500	500	5	30,000 h^−1^	110–300	>90%	Slowly deactivated with 100 ppm SO_2_ at 180 °C	[302]
Mn-TNTs@Ce	500	500	5	30,000 h^−1^	130–375	>90%	Relatively stable NO conversion with 5 % H_2_O and 100 ppm SO_2_ at 200 °C	[303]
H-MnO_2_@TiO_2_@HL	500	500	5	30,000 h^−1^	175–260	>90%	Reversible inhibition with 5% H_2_O at 180 °C	[294]
Ce@Mn@TiO_x_	1000	1000	5	30,000 h^−1^	140–230	>90%	Inhibition with 5% H_2_O at low temperature	[304]
MnCeO_x_@ZSM-5	500	500	5	960,000 mL·g^−1^·h^−1^	220–380	>90%	Relatively stable NO conversion with 5% H_2_O and 100 ppm SO_2_ at 300 °C	[305]
Fe_2_O_3_@MnO_x_@CNT	550	550	5	20,000 h^−1^	120–300	>90%	Reversible inhibition with 10% H_2_O and 100 ppm SO_2_ at 180 °C	[306]
mesoTiO_2_@MnCe/CNTs	500	500	3	10,000 h^−1^	225–400	>90%	Relatively stable NO conversion with 200 ppm SO_2_ at 300 °C	[307]
Co_(3-x)_Mn_x_O_4_@TiO_2_	500	500	5	24,000 h^−1^	125–275	>80%	Relatively stable NO conversion with 10% H_2_O and 100 ppm SO_2_ at 225 °C	[308]

^1^ TNT: titanium nanotube; TNTs: titanium nanotubes; layered double oxide; HL: hydrophobic layer; CNT: carbon nanotube; CNTs: carbon nanotubes.

**Table 7:** NH_3_-SCR activity in the presence of H_2_O and/or SO_2_ over some Ce-based catalysts.

Catalysts ^1^	Reaction Conditions						NO_x_ Conversion (%)	Effects of H_2_O/SO_2_	Ref.
	NO (ppm)	NH_3_ (ppm)	O_2_ (vol%)	H_2_O (vol%)	GHSV	T (°C)			
CO-pretreated CeO_2_	600	600	5	-	108,000 h^−1^	250–350	>90%	Stable NO conversion with 5% H_2_O and 100 ppm SO_2_ at 200 °C	[338]
H_2_SO_4_-pretreated CeO_2_	500	500	5	-	60,000 mL·g^−1^·h^−1^	350–475	>90%	Stable NO conversion with 5% H_2_O and 100 ppm SO_2_ at 400 °C	[339]
CeO_2_/HAT	500	500	5	-	177,000 h^−1^	250–400	>90%	Stable NO conversion with 5% H_2_O and 50 ppm SO_2_ at 300 °C	[340]
TiO_x_/CeO_2_	500	500	5	-	90,000 h^−1^	250–450	>80%	Irreversible deactivation with 5% H_2_O and 200 ppm SO_2_ at 300 °C	[341]
CeO_x_/TiO_2_	500	500	5	-	90,000 h^−1^	250–450	>80%	Irreversible deactivation with 5% H_2_O and 200 ppm SO_2_ at 300 °C	[341]
CeTiO_x_ Hollow nanotube	1000	1000	3	-	40,000 h^−1^	180–390	>98%	Stable NO conversion with 6% H_2_O and 100 ppm SO_2_ at 240 °C	[342]
CeO_2_-TiO_2_/P25	500	500	5	5	60,000 mL·g^−1^·h^−1^	300–450	>80%	Stable NO conversion with 5% H_2_O and 100 ppm SO_2_ at 400 °C	[343]
CeTiO_x_	600	600	3	-	150,000 h^−1^	180–300	>80%	Slowly deactivated with 5% H_2_O and 100 ppm SO_2_ at 225 °C	[344]
F-Ce-Ti oxides	500	600	5	-	41,000 h^−1^	180–240	>90%	Reversible deactivation with 100 ppm SO_2_ at 150 °C	[345]
P-CeO_2_/TiO_2_	500	500	5	-	60,000 h^−1^	200–430	>80%	Relatively stable NO conversion with 5% H_2_O and 500 ppm SO_2_ at 200 °C	[346]
Ce/Ti-Si-Al oxides	500	500	5	-	65,000 h^−1^	215–465	>80%	Reversible deactivation with 10% H_2_O and 100 ppm SO_2_ at 320 °C	[347]
Ce/TiO_2_-SiO_2_	500	500	3	-	28,000 h^−1^	250–450	>90%	100% NO_x_ conversion for 24 h with 10% H_2_O and 200 ppm SO_2_ at 300 °C	[348]
Ce/Mo-TiO_2_	500	500	5	-	60,000 mL·g^−1^·h^−1^	200–350	~100%	Stable NO conversion with 2% H_2_O and 100 ppm SO_2_ at 250 °C	[349]
WO_3_/CeO_2_	500	500	3.5	-	60,000 mL·g^−1^·h^−1^	250–450	~100%	Slowly deactivated with 4% H_2_O and 300 ppm SO_2_ at 200 °C	[350]
Ce-W/UiO-66	300	300	3	-	10,000 h^−1^	200–350	>90%	Stable NO conversion with 5% H_2_O and 200 ppm SO_2_ at 250 °C	[351]
Ti-Sn-Ce-O_x_	600	600	6	-	20,000 h^−1^	200–375	>90%	Stable NO conversion with 10% H_2_O and 300 ppm SO_2_ at 200 °C	[352]
CeBi/TiO_2_	600	600	5	-	108,000 h^−1^	250–400	>95%	Relatively stable NO conversion with 5% H_2_O and 100 ppm SO_2_ at 150 °C	[353]
CeSnO_x_/TiO_2_	500	500	5	-	30,000 h^−1^	200–420	>90%	Relatively stable NO conversion with 5% H_2_O and 100 ppm SO_2_ at 220 °C	[354]
Sn-Ce-Ti oxides	500	500	5	-	30,000 h^−1^	180–460	>90%	Stable NO conversion with 300 ppm SO_2_ at 240 °C	[355]
CuSO_4_-CeSnTiO_x_	500	500	5	-	30,000 h^−1^	240–340	>90%	Relatively stable NO conversion with of 5% H_2_O and 100 ppm SO_2_ at 240 °C	[356]
Co-Ce-Ti oxides	500	500	5	-	30,000 h^−1^	200–440	~100%	Slowly deactivated with 5% H_2_O and 100 ppm SO_2_ at 180 °C	[357]
Ge-Ce-W oxides	1000	1000	2	-	50,000 h^−1^	200–470	>95%	Deactivation with 100 ppm SO_2_ at 220 °C	[358]
Ce–Nb oxides	650	650	5	-	120,000 mL·g^−1^·h^−1^	220–400	~100%	Stable NO conversion with 5% H_2_O and 50 ppm SO_2_ at 280 °C	[359]
Ce_0.4_Nb_0.6_ nanospheres	500	500	5	-	30,000 h^−1^	250–450	>98%	Reversible deactivation with 200 ppm SO_2_ at 300 °C	[360]
Ce_20_Nb_20_Ti oxides	1000	1000	3	-	90,000 h^−1^	250–460	>95%	Deactivated with 10% H_2_O and 200 ppm SO_2_ at 350 °C	[361]
Ce-Nb-Ti oxides	1000	1000	3	-	90,000 h^−1^	250–500	>90%	Irreversible deactivation with 500 ppm SO_2_	[362]
Nb/CeSi_2_	500	500	4	-	60,000 h^−1^	215–450	>80%	Relatively stable NO conversion with 5% H_2_O and 200 ppm SO_2_ at 250 °C	[363]
Ni-Ce-La oxides	1000	1000	5	-	20,000 h^−1^	270–390	>90%	Stable NO conversion with 10% H_2_O and 500 ppm SO_2_ at 300 °C	[364]
Mo-CeO_2_/TiO_2_	500	500	5	-	128,000 h^−1^	275–400	>90%	Relatively stable NO conversion with 5% H_2_O and 50 ppm SO_2_ at 300 °C	[365]
Mo_0.1_CeSi_2_	500	500	5	-	90,000 h^−1^	215–400	>90%	100% NO_x_ conversion for10 h with 5% H_2_O and 200 ppm SO_2_ at 250 °C	[366]
Cr-Ce-Ti oxides	500	500	5	-	40,000 h^−1^	182–405	>90%	Slowly deactivated with 5% H_2_O and 100 ppm SO_2_ at 250 °C	[367]
Cr_1_CeZr_2_O_x_	600	600	5	5	108,000 h^−1^	200–350	>90%	Stable NO conversion with 5% H_2_O and 100 ppm SO_2_ at 250 °C	[368]
Zr-CeTiO_x_	500	500	5	-	60,000 h^−1^	200–375	>95%	Stable NO conversion with 5% H_2_O and 50 ppm SO_2_ at 225 °C	[369]
P-Ce-Zr-Ti oxides	800	720	3	-	30,000 h^−1^	180–400	>80%	Relatively stable NO conversion with 5% H_2_O and 200 ppm SO_2_ at 300 °C	[370]
Ce-Sn-W-Ba oxides/TiO_2_	930	930	10	-	8000 h^−1^	235–470	>90%	Relatively stable NO conversion with 5% H_2_O and 200 ppm SO_2_ at 350 °C	[371]
CeO_2_-WO_3_-palygorskite/TiO_2_	500	500	5	-	30,000 h^−1^	240–400	>80%	Relatively stable NO_x_ conversion with 100 ppm SO_2_ at 360 °C	[372]
Sulfated CeO_2_-Rod	500	500	3	-	150,000 mL·g^−1^·h^−1^	300–450	~100%	Stable NO_x_ conversion with 5% H_2_O and 100 ppm SO_2_ at 250 °C	[373]
Sulfated CeO_2_-ZrO_2_	250	250	2.5	-	90,000 h^−1^	250	90%	Relatively stable NO_x_ conversion with 200 ppm SO_2_ at 250 °C	[374]
CeO_2_@TiO_2_ Core-shell	500	500	5	-	24,000 h^−1^	200–450	>80%	Irreversible deactivation with 200 ppm SO_2_ at 280 °C	[375]
CeO_2_@TiO_2_	500	500	5	-	36,000 h^−1^	225–400	>95%	Relatively stable with 5% H_2_O and 100 ppm SO_2_ resistance at 350 °C	[376]

^1^ HAT: halloysite.

## Data Availability

The original contributions presented in the study are included in the article; further inquiries can be directed to the corresponding author.
